# A large-scale evaluation of commonsense knowledge in humans and large language models

**DOI:** 10.1093/pnasnexus/pgag029

**Published:** 2026-02-16

**Authors:** Tuan Dung Nguyen, Duncan J Watts, Mark E Whiting

**Affiliations:** Department of Computer and Information Science, University of Pennsylvania, Philadelphia, PA 19104, USA; Department of Computer and Information Science, University of Pennsylvania, Philadelphia, PA 19104, USA; Operations, Information and Decisions Department, The Wharton School, University of Pennsylvania, Philadelphia, PA 19104, USA; Annenberg School for Communication, University of Pennsylvania, Philadelphia, PA 19104, USA; Department of Computer and Information Science, University of Pennsylvania, Philadelphia, PA 19104, USA; Operations, Information and Decisions Department, The Wharton School, University of Pennsylvania, Philadelphia, PA 19104, USA; Pareto, San Francisco, CA 94111, USA

**Keywords:** commonsense knowledge, AI, large language models, crowdsourcing

## Abstract

Commonsense knowledge, a major constituent of AI, is primarily evaluated in practice by human-prescribed ground-truth labels. An important, albeit implicit, assumption of these labels is that they accurately capture what any human would think, effectively treating human common sense as homogeneous. However, recent empirical work has shown that humans vary enormously in what they consider commonsensical; thus what appears self-evident to one benchmark designer may not be so to another. Here, we propose a method for assessing commonsense knowledge in AI, specifically in large language models (LLMs) that incorporates empirically observed heterogeneity among humans by measuring the correspondence between a model’s judgment and that of a human population. We first find that, when treated as independent survey respondents, most LLMs remain below the human median in their individual commonsense competence. Second, when used as simulators of a hypothetical population, LLMs correlate with real humans only modestly in the extent to which they agree on the same set of statements. In both cases, smaller, open-weight models are surprisingly more competitive than larger, proprietary frontier models. Our evaluation framework, which ties commonsense knowledge to its cultural basis, contributes to the growing call for adapting AI models to human collectivities that possess different, often incompatible, social stocks of knowledge.

Significance Statement Commonsense knowledge is an important component of human-like AI, of which large language models (LLMs) are a notable example. However, evaluating this knowledge is challenging because different benchmark designers can have extremely conflicting intuitions about what sensible “ground truths” are. We address this by proposing a new LLM evaluation method based on empirical correspondence with real humans on a large scale. Our results show that the variation in human common sense is far from fully captured by LLMs, either as independent agents or as simulators of a synthetic population. Finally, we argue that explicitly capturing the heterogeneity in human commonsense judgments is crucial to developing AI models that will better interact with real humans in diverse social contexts.

## Introduction

The physical and social worlds are tremendously complex and unpredictable; yet, humans are able to navigate these environments almost effortlessly thanks to a special aptitude called common sense (1). Endowing machines with this ability has remained a grand challenge throughout the history of AI research (2–6). Recently, significant progress was made by large language models (LLMs), a class of machine learning systems that can synthesize commonsense knowledge extensively from their training data (7–11), make highly flexible generalizations (12), and thus exhibit increasingly human-like tendencies (13–17).

**Fig. 1.**
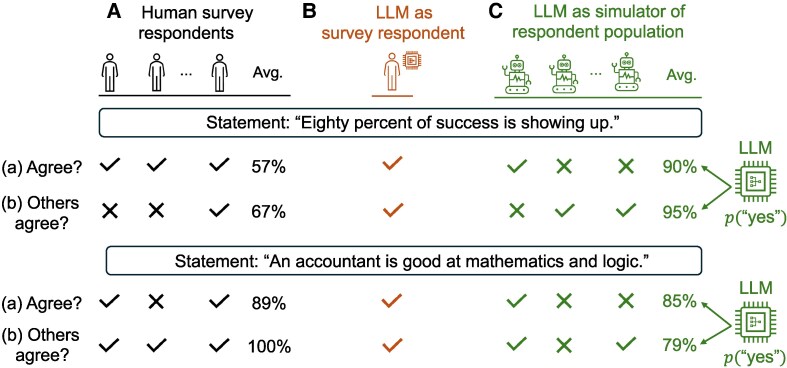
Evaluation settings to measure the common sense of humans and LLMs. For every statement, humans and LLMs are asked to indicate (a) whether they agree with it and (b) whether they think most other people would agree with it. In A), a total of N=2,046 human participants were recruited to perform this task. The “Avg.” column denotes the percentage of people who answered “yes” to the corresponding question. In B), we treat each LLM (in a total of N=35 models) as an independent survey respondent, just like every human in panel A. This gives rise to the *individual*-level view of common sense, in which this model is measured based on its agreement with the majority of other people on every statement. In C), we treat every LLM’s probability in its output answer as the average response of a hypothetical population of “silicon samples” (depicted as robots). For instance, if the LLM agrees with the statement “Eighty percent of success is showing up” with 90% probability, we interpret this as 90% of the silicon samples would agree with this statement. This gives rise to the *statement*-level metric of common sense, which is used to measure the correlation between the human (panel A) and silicon sample (panel C) populations.

Progress in LLM common sense is frequently evaluated by standardized benchmarks (18). While their details vary, most existing benchmarks are conceptualized around the notion of *correctness*: they assume humans apprehend matters of everyday reality in a uniform manner, and hence assess models by how accurately they recognize this “ground truth.” However, what different individuals may hold as trivial, commonsensical truths necessarily vary because their experience of the world is often highly subjective (19–23). Empirical research corroborates this view, showing that humans are extremely heterogeneous in their judgment even of simple, seemingly obvious propositions (24). For instance, when asked to evaluate the aphorism “Eighty percent of success is showing up,” illustrated in Fig. 1A, nearly half of the human respondents actually disagreed with it.

Recent audits of AI commonsense benchmarks have found that ground-truth labels often achieve low agreement among independent data annotators, and up to a quarter of them are even contradicted after relabeling (25). Thus, when an LLM achieves a high accuracy score, it appears *only* similar to its benchmark designer. To another person who prescribes a vastly different set of ground truths—according to their own common sense—the same model could appear just as dubious. The challenge to evaluation, then, is in reconciling the aim for an informative metric with the pluralism in human judgment that permits almost no correct labels.

In the human sciences, common sense is treated not as a set of irrefutable truths, but rather as a system of beliefs that are mutually upheld by members of a social community (26). In other words, a person has common sense only if their beliefs coincide with whatever their community holds collectively. This conception allows for an empirical investigation of commonsense knowledge, and thus motivates our contribution of a new LLM evaluation framework. We start with a population—of humans and of LLMs alike—and collect their judgments toward a number of statements, from which consensus arises. Common sense is then measured by the degree to which members of this population agree with one another about these statements. We elaborate this notion of *collective agreement* via two major but logically independent uses of an LLM, illustrated in Fig. 1.

First, an LLM can be viewed as an independent survey respondent that is evaluated on an individual basis (Fig. 1B). Under this role-playing paradigm (27, 28), the respondent must both subjectively agree with the majority opinion of other people (Fig. 1A), and accurately predict this majority opinion regardless of what it subjectively holds. These two signals are combined to measure the commonsense competence of the LLM respondent, just like how a human would be scored. The results show that both humans (N=2,046) and models (N=35) vary significantly in this respect. While the highest-ranked model is rated as commonsensical as 64.5% of recruited human participants, over two-thirds of LLMs are placed below the human median. Surprisingly, we find that smaller, open-weight models like Mistral-7B and Flan-T5-XXL are in fact comparable or even more competitive than larger, proprietary models like GPT-4/5 or Claude 3 Opus.

Second, an LLM can be viewed as a summarizer of social and cultural knowledge (29, 30) and thus be evaluated by how well it reproduces the distribution of human beliefs via simulating a hypothetical collective (31–37). In such a population, “silicon samples” (depicted as robots in Fig. 1C) provide ratings just like humans, and common sense is measured for each statement based on how widely agreed upon it is. Between the two populations in Fig. 1A and C, we find that commonsense scores for the same set of statements are modestly correlated (Pearson’s *r* up to 0.43), but this relationship remains significantly below the human internal reliability (r=0.60). Some groups of silicon samples, moreover, exhibit traceable qualitative differences from humans; for instance, the population constructed by Gemini Pro 1.0 overwhelmingly associates common sense with figures of speech, while humans tend to see eye to eye much more often on simpler, literal expressions.

Current practices in AI commonsense evaluation have been subjected to several criticisms. For example, as a product of large-scale crowdsourcing efforts, benchmarks often contain noisy human annotations (38) or semantically incoherent stimuli (39), thereby casting doubt on the reliability of their performance metrics. Our present argument, however, is that not only can humans be noisy in their judgments, they may also hold genuinely conflicting beliefs about what is self-evident. Developing human-like AI requires explicitly acknowledging this pluralism, incorporating it in benchmarking domains where ground-truth labels likely do not exist, and appropriately defining what human performance is (6, 25). We address these concerns by contributing a framework to evaluate commonsense knowledge in LLMs that is grounded in its social basis, ie in correspondence with the judgments of a human group surveyed on a large scale. From an AI alignment perspective, our bottom–up analysis allows for a fine-grained, empirical assessment of the content of commonsense knowledge represented in intelligent machines, especially when they are situated in highly diverse social contexts where questions of cultural awareness often arise (40).

## Overview

We use a dataset introduced in Ref. (24) which contains N=4,407 statements in English taken from seven sources, including two AI corpora and two types of direct elicitation by online participants. Humans (N=2,046) were independently recruited on Amazon Mechanical Turk to rate these statements. The Methods section and SI Appendix, Table S1 describe this human sample in more detail. Participants were shown one statement at a time and asked to indicate (a) whether they agreed with it and (b) whether they thought most other people would agree with it. For example, in Fig. 1A, 89% of humans agreed with the statement “An accountant is good at mathematics and logic,” while 100% of the same people believed that most others would agree with it. Each participant was assigned 50 statements selected at random, and on average every statement was labeled by 23 people. We determine a statement’s human majority rating—agree or disagree with it, in response to question (a)—as the judgment that was held by at least half of those who were assigned to rate it (see the Methods section and SI Appendix, Section A).

We examine a total of 35 autoregressive LLMs including 10 proprietary, frontier models such as GPT-3.5/4/5, Claude 3, Gemini Pro 1.0, and Mistral-Large, as well as 25 open-weight models such as LLaMA-2/3 and Falcon. Among the open-weight LLMs, the smallest model has 80M parameters, while the largest has 180B. The full list can be found in SI Appendix, Table S3. For each statement, we prompt a model with the same questions (a) and (b) above and record the probabilities with which it generates the tokens “yes” and “no” (depicted in the “Avg.” column in Fig. 1C). When binary answers are called for—such as in Fig. 1B where the LLM is treated as an individual agent—we choose the answer that is associated with the higher probability. Otherwise, these probabilities are interpreted as the frequencies with which the hypothetical silicon samples (Fig. 1C) would answer the same questions with a “yes” or “no.” The Methods section and SI Appendix, Section B provide more information and precise definitions. SI Appendix, Figures S4 and S5 further evaluate the calibration between a model’s probability and the frequency of the same response among humans for several models.

## LLMs as independent survey respondents

Upon its release, GPT-4 was demonstrated to score above 90% of the Uniform Bar Examination takers (41). Notably, this evaluation is unambiguous for exam questions are designed to have correct answers—and humans and machines can be objectively measured by how accurately they select such answers. Unfortunately, as we have argued, this does not apply to common sense: what appears correct to someone may not be so to another.

**Fig. 2.**
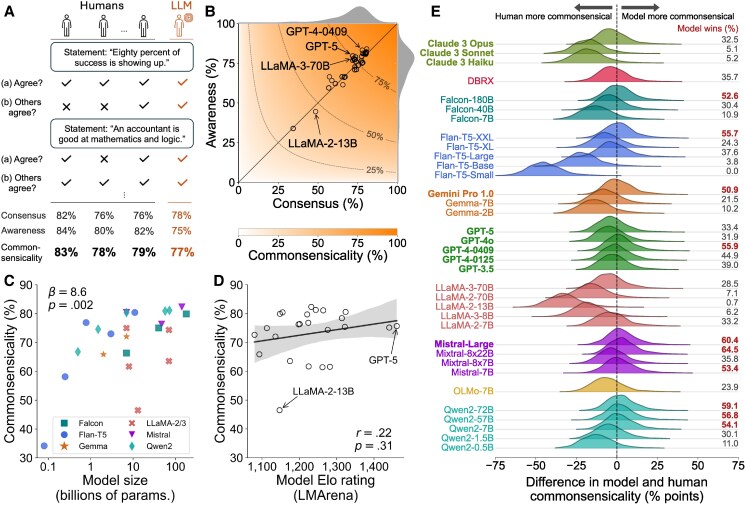
Individual-level commonsensicality of large language models. A) The image shows conceptually how individual commonsensicality, defined for every human and model, is calculated based on their judgments of each candidate statement. B) The image shows each model’s consensus and awareness scores. The level curves depict combinations of consensus and awareness that produce three different values of commonsensicality scores: 25, 50, and 75%. C) The image shows the relationship between a model’s commonsensicality and its size, measured by the number of trainable parameters. Here, we only select six model families that each have at least two models of which we know the sizes. Also illustrated are the regression coefficient (*β*) and its two-sided *P*-value, estimated using a linear mixed-effect model predicting commonsensicality using an LLM’s (log-)size, grouped by model family, such as Flan-T5. D) The image shows the relationship between a model’s commonsensicality and its Elo rating on the LMArena benchmark. Only 24 models with an Elo rating are shown in this figure. Pearson correlation *r* and its two-sided *P*-value are displayed. Correlation is also displayed by the best-fit line and a 95% CI for the regression estimate (using 1,000 bootstrapped samples). E) The image compares commonsensicality between humans and LLMs. The *x*-axis represents the percentage-point difference in commonsensicality between a model and a person, where a positive difference indicates the model is more commonsensical. The *y*-axis represents the kernel density of this difference. The “model wins” column to the right is the frequency with which a model is judged more commonsensical than a person, which equals the area under the density curve to the right of the vertical dashed line at 0. Closed-source models’ names are in bold.

Here, we describe an alternative LLM evaluation strategy, previously illustrated in Fig. 1B and now in more detail in Fig. 2A, that can overcome this ground-truth deficiency. Since there is no guarantee of a correct answer, the common sense of humans and models is simply measured by how much they agree with one another. In particular, we view each LLM as an independent survey respondent who, like every other human participant, provides binary ratings for every statement. The model’s common sense is determined via two signals. First, does its judgment of a statement, via question (a), coincide with the human majority rating? Second, is its prediction of the human majority, via question (b), correct? Averaged over all N=4,407 statements, these signals respectively give us the model’s *consensus* and *awareness* scores. We take the geometric average of these two scores and call the result the model’s *commonsensicality*. Figure 2B presents these three scores for all 35 models. See the Methods section and SI Appendix, Sections A and C for definitions and Table S5 for precise figures.

The notions of consensus and awareness are rooted in the functional analysis of common sense in sociology (42). More specifically, in order for members of a social group to meaningfully interact with one another, a baseline in the form of a shared body of knowledge—what they each know about the ordinary life they live in and share with others—must be achieved among them. Consensus is intended to measure this “shared” aspect. Whenever necessary in the course of social interaction, moreover, the same people must also be able to appeal to this shared knowledge and receive appropriate response from others. This requires not only knowing something but also knowing that other people share that knowledge. This is what awareness aims to capture.

A model with high commonsensicality must both agree with the human majority (leading to high consensus) and accurately predict what most people think, regardless of its own judgment (high awareness). For example, in Fig. 2A, the statement “Eighty percent of success is showing up” received ratings from 21 human participants, 12 of whom (57%) agreed with it. To have high commonsensicality, the model is expected both to agree with this statement and to predict that most people would agree with it. At one extreme, we have a maximum score of 100%, where this always happens. A lower commonsensicality score could be driven by a model’s lower consensus, awareness, or both. For instance, Claude 3 Opus disagrees with this statement, thereby lowering its consensus score, but it correctly predicts that most people would agree with it, thereby raising its awareness score. On average, this kind of divergence in a model’s answers to questions (a) and (b) happens about 10% of the time, mostly in response to aphorisms; further example statements can be found in SI Appendix, Table S4.

The commonsensicality score achieves two important goals. First, it requires no prior ground truth for any candidate statement: what is “true” is entirely determined by what people articulate as true. Second, the commonsensicality score subjects humans and LLMs to the same calculation, for they perform the same rating task; this allows us to make empirically commensurable comparisons between them, as will be shown shortly. Our metric thus bears resemblance to the concept of cultural competence (43) or cultural consonance (44)—the propensity of an individual to know what their society collectively deems appropriate—which often exhibits significant heterogeneity among that society’s members. Note, however, that the majority vote as used here is based solely on human judgments, and thus humans may have a “head start” when compared to LLMs under this metric. SI Appendix, Section C.5 introduces an alternative formulation addressing this concern and shows that the results reported here remain robust under this different setting.

We observe in Fig. 2B that most models lie close to the diagonal line, implying their consensus and awareness are roughly equal. This is largely because these models almost always give the same answer to questions (a) and (b). All three scores are left-skewed in Fig. 2B; for instance, the average commonsensicality score among models is 71.9% (SD=10.6 percentage points). The most commonsensical LLM, with a score of 82.3%, is Mixtral-8x22B, Mistral AI’s open-weight model based on the mixture-of-expert architecture with 141B parameters. Other top-performing models include both open- and closed-source LLMs such as Mistral-Large (81.3%, closed), Qwen2 (72B: 81.1%, 57B: 80.9%, open), GPT-4 (80.6%, 0409 version, closed), and Flan-T5-XXL (80.4%, open). The least commonsensical models also include open-weight and closed-source models such as Flan-T5 (Small: 34.2%, Base: 58.1%, open), LLaMA-2-13B (46.5%, open), and Claude 3 (Haiku: 61.4%, Sonnet: 61.6%, closed).

It is generally observed that within the same LLM family (such as LLaMA), a model’s benchmarking performance tends to increase with its size, or number of trainable parameters—a phenomenon dubbed the “scaling law” (45). We also find the same pattern on this commonsensicality test. Figure 2C depicts this relationship for 23 models belonging zo six families in our collection, each of which contains at least two models whose sizes are known. As can be noticed, this correlation is the most pronounced within the Flan-T5 and the least within the Mistral families. It should be noted, however, that the scaling law does not only apply to model size; another significant factor is the quantity and quality of their training data, which might explain the large variance in commonsensicality among models of roughly 7 billion parameters in the LLaMA-2/3, Qwen2, and Gemma families. To quantify this relationship while accounting for heterogeneity across model families, we fit a mixed-effect regression model with commonsensicality as the outcome, the logarithm of a model’s size as the fixed effect, and the model family as the random effect. The result reveals that, on average, a 10-fold increase in an LLM’s size is associated with an 8.6 percentage-point increase in its commonsensicality score (P=0.002, 95% CI [3.4–13.4]; see the Methods section and SI Appendix, Section C.1).

Interestingly, an LLM’s commonsensicality does not seem to correlate with its general appeal to humans. Figure 2D depicts the relationship between a model’s commonsensicality and its Elo rating on the LMArena benchmark (46). This rating is calculated based on human preference signals, where online users interacted with a pair of randomly chosen models and decided which one they preferred. We choose this benchmark because it is based on realistic, on-the-fly interactions between LLMs and humans, which likely involve a lot of everyday commonsense reasoning unlike other static benchmarks. If a model scores 100 points higher than another, then the former is preferred to the latter about 64% of the time. See the Methods section and SI Appendix, Section C.2 for more details. Surprisingly, we do not find any significant correlational evidence between the commonsensicality and Elo scores; Pearson’s r(22)=0.22, P=0.31, 95% CI [−0.21 to 0.57]. With a large gap in Elo rating of 90, for example, GPT-4-0125 is preferred to GPT-3.5 about 63% of the time by humans when pitched side by side. Yet their commonsensicality scores are very similar, at 78.4 and 76.8%, respectively. In SI Appendix, Section C.3, we perform this analysis with respect to an alternative commonsense reasoning benchmarking result (47) and make the same observation.

Like LLMs, humans also vary considerably in their individual commonsensicality; SI Appendix, Fig. S2 presents the same version of Fig. 2B but for humans. Thus, it would be unreasonable to accept a singular level of “human performance” to which LLMs can be compared—an assumption made by most existing AI benchmarks. In light of this fact, we instead examine where exactly in this distribution of human commonsensicality scores each LLM is positioned. Note that during data collection, every human respondent was only tasked with labeling a random subset of 50 statements, whereas each model manages to label all 4,407 of them. For fairness, when comparing each LLM with every human participant, we restrict the calculation of commonsensicality to the subset of 50 statements that the participant was asked to label. The Methods section and SI Appendix, Sections A and C provide more detail on this calculation.

Figure 2E presents the result of this comparison. The *x*-axis is the percentage-point difference between a model’s and a human’s commonsensicality scores; a positive difference indicates that the model is more commonsensical than the human. The *y*-axis depicts the estimated density of this difference across all 2,046 humans. Therefore, the area under this curve, from 0 onward, represents the model’s percentile in the distribution of human commonsensicality scores, which is also reported in the “model wins” column.

Relative to humans, most LLMs are modest in their individual-level common sense. Over two-thirds of models (25 out of 35) are placed below the human median, meaning they would be judged less competent than a participant chosen uniformly at random. For instance, Claude 3 Opus, a frontier model, is judged as competent as only about a third of humans. The rest of these LLMs, which are indeed above the human median, include both closed-source models like GPT-4 (0409 version), Gemini Pro 1.0, and Mistral-Large, and open-weight models such as Falcon-180B, Mixtral-8x22B, and Flan-T5-XXL. The highest-ranked model according to this metric is Mixtral-8x22B, which is rated above 64.5% of human participants. Most surprisingly, Flan-T5-XXL is an LLM that was probably trained on orders of magnitude *less* data than today’s models, yet it is comparable to GPT-4-0409 and even ranked higher than Falcon-180B, a model that is about 16 times its size.

The LLaMA family is also a notable case. No LLM within this family manages to be more commonsensical than a third of humans. Moreover, even though the scaling law is observed in other model families, this is not the case for LLaMA-2. The highest-scoring model turns out to be the smallest variant, LLaMA-2-7B, with a winning rate of 33.2%. This rate drops to almost zero for the 13B version before coming back to 7.1% for the 70B variant. This observation is not even consistent with the inverse scaling law (48, 49), where models are expected to be *worse* in some metric as they grow in size. Although without further evidence, we suspect that the creation of LLaMA-2 models may not have been uniform, especially in terms of the data used to train them.

In summary, the commonsensicality score presented here measures an LLM’s commonsense competence with respect to how often it agrees with humans. Unlike existing AI benchmarks, this evaluation is bottom–up: it is derived from the consensus of real humans as opposed to a researcher-prescribed correct label. Our metric can therefore be flexibly adapted to any other population of interest to analyze an LLM *relative* to that population. Moreover, the commonsensicality metric formalizes the notion of human performance which, as we discussed above, displays a nontrivial variation. This is also in contrast with current benchmarks, as human performance is often thought to be constant and (close to) perfect. As a result, we do not interpret commonsense intelligence as an LLM’s ability to pass a rigid, predefined accuracy threshold, but rather analyze this model by locating it in the heterogeneity of human competencies. Based on this, we have shown that smaller, open-weight models can be as competitive as their larger, proprietary, frontier counterparts.

## LLMs as aggregators of human opinion

Recently, a growing body of work in the social sciences has demonstrated that the distribution of human opinions and attitudes in many empirical surveys can be approximated surprisingly well by the same answers sampled from LLMs. This includes quantities such as public opinion (32, 37, 50, 51), voting preferences (31), social conventions (52), moral judgments (33), and economic decisions (53, 54). In explaining this remarkable correspondence, several authors have argued that since so many human individuals have contributed their microlevel knowledge to the LLMs’ training data, these models can be viewed as a “distilled form of crowdsourcing” (55) capable of reproducing the diversity of this knowledge with great fidelity (36, 56, 57).

We apply this perspective to the evaluation of LLM common sense, making two running assumptions. First, similar to voting preferences and moral judgments, cultural artifacts such as commonsense knowledge are also widely captured in LLMs’ training data. And second, throughout training LLMs can effectively synthesize these artifacts in a way that represents the average human contributor of that data. Simply put, the more frequently a statement is endorsed by humans in the data, the more likely LLMs are to accept it. The goal of this section is thus to examine how well LLMs, when used as a generator of a hypothetical population, can approximate human commonsense knowledge as articulated by the human participant pool.

**Fig. 3.**
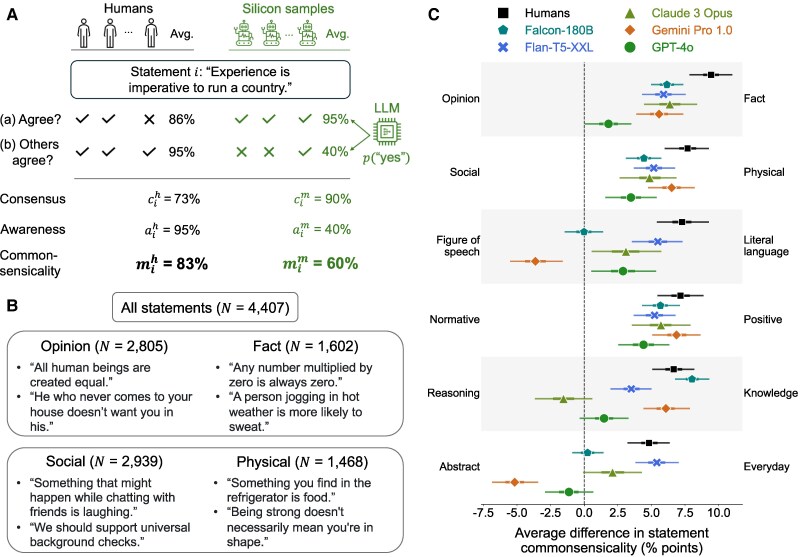
Commonsensicality of statements in different populations of raters. In A), we depict two populations: one consisting of real humans and one of silicon samples generated by repeatedly sampling the responses of GPT-3.5. For each population and every statement *i*, we measure two quantities: how close participants in that population are to a unanimous judgment of the statement (consensus, $c_{i}^{h}$ and $c_{i}^{m}$) and how well they can predict this majority opinion (awareness, $a_{i}^{h}$ and $a_{i}^{m}$). The statement’s commonsensicality, $m_{i}^{h}$ and $m_{i}^{m}$, is the geometric average of its consensus and awareness scores. For example, the statement “Experience is imperative to run a country” is $m_{i}^{h}=83\%$ commonsensical according to humans, but only $m_{i}^{m}=60\%$ commonsensical according to GPT-3.5-simulated silicon samples. B) The image illustrates some features of these statements in our corpus. Every statement is labeled as either an objective fact (N=1,602) or a subjective opinion (N=2,805); to describe either the physical world (N=1,468) or social reality (N=2,939). In total, there are six such dichotomies, which are described further in the Methods section and SI Appendix, Section D. C) The image shows the difference in statement commonsensicality score within each dichotomy for several populations of raters. For instance, in the human population, each statement *i* receives a commonsensicality score of $m_{i}^{h}$. The black square in the top row represents the average difference in this score between statements labeled as a fact and those labeled as an opinion. Therefore, to humans, facts are on average 9.38 points more commonsensical than statements. Thick and thin bars depict the 50 and 95% CI from 1,000 bootstraps.

Imagine a hypothetical society of “silicon samples” (31), previously illustrated in Fig. 1C and now in more detail in Fig. 3A. For each statement, individuals in this society are tasked with answering the same questions (a) and (b) described in the Overview section. These answers are generated by repeatedly sampling an LLM’s response to the same prompt, taking advantage of its probabilistic nature. Assuming independence, the average response of a silicon sample in the limit is exactly the probability with which the LLM generates the “yes” token to a given question. Based on this signal, illustrated in the “Avg.” column of Fig. 3A, we define a *commonsensicality* score for the statement. Essentially, a higher score indicates that individuals in this population are close to unanimity in their judgment of this statement (thus increasing the statement’s consensus) and that they can accurately predict this judgment in one another (thus increasing its awareness). Precise calculations can be found in the Methods section and SI Appendix, Section D. Note that this *statement*-level measure is separate from the *individual*-level commonsensicality score (cf. Fig. 2A) that was the subject of the previous section.

Figure 3A illustrates an example statement (indexed by *i*): “Experience is imperative to run a country.” Of the 22 human participants who were assigned statement *i*, 19 (or 86%) agreed with it, while 21 (or 95%) believed other people would agree with it. This statement accordingly receives a consensus score of $c_{i}^{h}=73\%$, an awareness score of $a_{i}^{h}=95\%$, and a commonsensicality score of $m_{i}^{h}=83\%$; see the Methods section for the calculation. Thus, within this human group, statement *i* is 83% commonsensical. On the other hand, in the population of silicon samples generated by GPT-3.5, depicted by robots, 95% of them agree with this statement, while 40% of them express that others would agree with it. By the same calculation, we arrive at statement *i*’s commonsensicality among the GPT 3.5-generated population, which is $m_{i}^{m}=60\%$.

### Features of common sense in statements between humans and silicon samples

What makes two populations of raters (eg humans and silicon samples) similar in their common sense? We propose to analyze the commonsensicality scores for the same set of statements between these groups. First, we look at what types of statements tend to attract high agreement in each population. Our corpus is accompanied by six epistemological features: Each statement was rated to depict either an objective fact or a subjective opinion; to use either literal language or a figure of speech; to be about either an abstract rule or a description of an everyday experience; and so on. Figure 3B gives some example statements with respect to two features; the Methods section and SI Appendix, Section D provide a complete list as well as further examples.

For every population and each feature dichotomy, we compare the two groups of statements separated by that dichotomy with respect to their average commonsensicality scores. The results for humans and five silicon sample populations are shown in Figure 3C. For example, according to humans—represented by a black square at the top of the figure—statements rated as facts (eg “The Pope is the leader of the Catholic Church”) are on average 9.38 points more commonsensical than statements rated as opinions (eg “Never go on trips with anyone you do not love”); mean difference (MD) =9.38, 95% CI [7.72–10.93]. Among silicon samples generated by Falcon-180B (teal pentagon), this difference is 6.18 points (95% CI [4.86–7.45]).

Thus, Fig. 3C shows that human common sense exhibits very clear tendencies: it strongly favors facts over opinions, descriptions of physical over social realities, literal expressions over figures of speech, etc. When examining LLMs, we find that most of these tendencies are preserved by silicon samples. For instance, those generated by Flan-T5-XXL (blue crosses) are almost indistinguishable from humans across all six dichotomies.

The silicon samples simulated by Falcon-180B are also similar to humans except along two dimensions. First, this population displays no significant preference between statements that use literal language (eg “A cat doesn’t want to get wet”) and those employing a figure of speech (eg “Rudeness is the weak man’s imitation of strength”) (MD =0.03, 95% CI [−1.51 to 1.46]). Second, it also does not favor statements that depict ordinary, everyday experiences (eg “A grain of sand is very small”) over abstract rules of thumb or aphorisms (eg “Morality is just a concept that can change depending on the situation”) (MD =0.26, 95% CI [−0.87 to 1.51]).

The three most popular and capable closed-source LLMs—Claude 3 Opus, Gemini Pro 1.0 and GPT-4o—all exhibit the same tendencies with humans in four out of six dichotomies. For instance, all three models generate a community that rates statements describing the physical world (“You are likely to find a shirt in closet”) as significantly more commonsensical than those about social experience (“Justice without force is powerless, force without justice is tyrannical”). GPT-4o’s simulated population (green circle), however, does not differentiate between claims about everyday reality with abstract statements (MD =−1.11, 95% CI [−2.81 to 0.65]). Whereas Gemini Pro 1.0 (orange diamond), like humans, favors statements that contain simple declarative knowledge about the world (e.g. “Plants cannot survive without light”) over those that involve logical reasoning (e.g. “If we ask for someone else to explain things they will think about it against their own justification”), Claude 3 Opus (green triangle), and GPT-4o do not display such distinction.

More starkly, we observe that the population generated by Gemini Pro 1.0 significantly diverges from humans in two dichotomies of knowledge. According to this simulated group, statements that use a figure of speech (e.g. “The clash of ideas brings forth the spark of truth”) are on average 3.67 points (95% CI 1.76–5.57) more commonsensical than those that use more literal expressions (e.g. “A person doesn’t want a low paying job”). Similarly, abstract statements (e.g. “Our greatest misfortunes come to us from ourselves”) are 5.17 points (95% CI 3.36–6.78) more commonsensical than statements describing everyday reality (e.g. “The last thing you do when you take a shower is dry off”). Although the reason for these surprising findings is unclear, they may suggest a significant difference in Gemini Pro 1.0’s training strategy, such as involving an over-representation of abstract figures of speech in its training set.

### Correlation in statement commonsensicality between humans and silicon samples

We have seen in Fig. 3 that LLM-simulated populations display largely similar qualitative tendencies to humans. Here, we provide a quantitative analysis of this correspondence. More specifically, if the populations of silicon samples and humans are similar with respect to their common sense, then the commonsensicality score of each statement should be similar in both populations. The “fidelity” of an LLM, therefore, can be measured by the Pearson correlation between $m_{i}^{m}$ and $m_{i}^{h}$ for all statements *i*.

First, to establish a baseline for the internal consistency of human judgments—given their subjectivity—we perform a resampling analysis. We repeatedly split the full human sample (N=2,046) into two random halves, calculate statement commonsensicality scores for each half, and compute the correlation in these scores between the two. Even among humans, the agreement on statement commonsensicality scores only reaches a moderate level; mean correlation r=0.60 (95% CI from 1,000 repetitions: [0.58–0.61]).

**Fig. 4.**
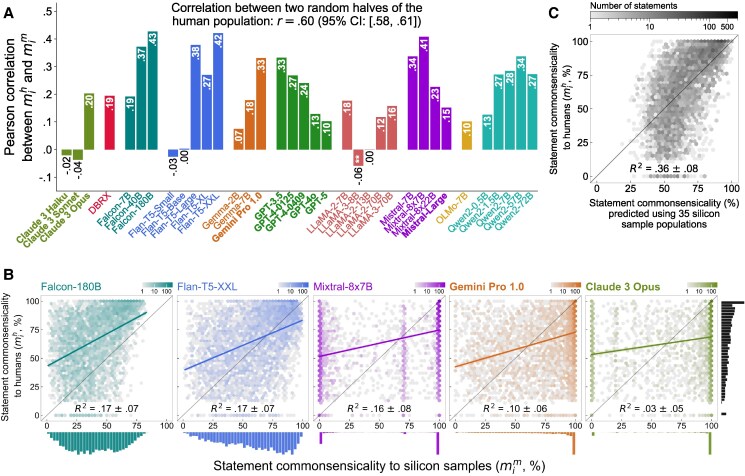
Correspondence between human and silicon sample populations with respect to statement scores. In each population, every statement receives a commonsensicality score. A) The image shows the Pearson correlation between $m_{i}^{h}$, the score in humans, and $m_{i}^{m}$, the score in silicon samples, for all models. All positive correlations are significant at the P<0.001 level. All negative correlations are insignificant with P>0.05, except for LLaMA-3-8B (P=0.003, depicted with “**” in the figure). *P*-values are two-sided and Bonferroni corrected. As a baseline, we also display the same correlation between two randomly split subpopulations of humans, which is r=0.60. The 95% CI is derived from 1,000 repetitions of such splits. B) The image expands this correlation for some models. The shade of each hexagon represents its density, i.e. the number of statements within that hexagon. We also illustrate a best-fit line in each plot, predicting $m_{i}^{h}$ with $m_{i}^{m}$ using a linear regression model. The out-of-sample $R^{2}$ for this model (mean and SD) is calculated using 50-fold cross-validation. In C), we combine the statement commonsensicality scores $m_{i}^{m}$ in *all* 35 silicon sample populations to predict the same score in humans, $m_{i}^{h}$, using a multiple regression model. We also report the out-of-sample $R^{2}$ for this model (mean and SD), calculated using 50-fold cross-validation.

Figure 4A shows the correlation in statement commonsensicality scores between humans and silicon samples generated by every model in our collection. The most faithfully representative models—Falcon-180B, Flan-T5-XXL, and Mixtral-8x7B—correlate with people at the r=0.41 to 0.43 level, well below the baseline of r=0.60. Unsurprisingly, for some model families like Falcon and Gemini/Gemma, larger or newer models tend to be more representative. For instance, the correlation coefficient rises from 0.19 to 0.37 and 0.43 for Falcon-7B, 40B, and 180B, respectively. On the other hand, for some families like GPT-3.5/4 and Mistral, the opposite is observed. For example, the correlation decreases from 0.33 for the earliest model, GPT-3.5, down to 0.13 for the latest variant, GPT-4o. One model, LLaMA-3-8B, has a negative correlation with humans (r=−0.06, P=0.003), while five models (Claude 3 Haiku, Claude 3 Sonnet, Flan-T5-Small, Flan-T5-Base, and LLaMA-2-13B) demonstrate no significant correlation at all.

Five of these correlations are magnified in Fig. 4B, from which we report two important observations. First, a considerable amount of variation in statement commonsensicality in humans ($m_{i}^{h}$) is not accounted for by the same score emerging from silicon samples ($m_{i}^{m}$). Using a linear model to regress $m_{i}^{h}$ via $m_{i}^{m}$ as the predictor, we depict the best-fit lines as well as their out-of-sample coefficients of determination ($R^{2}$) here. Only up to 17% of the variation in statement commonsensicality score according to humans can be explained, which is the case for Falcon-180B and Flan-T5-XXL. This figure is 10, 3, and 1% for Gemini Pro 1.0, Claude 3 Opus, and GPT-4o, respectively (the last of which is not shown in Fig. 3B).^a^

Second, the best-fit lines, as well as the marginal distributions in Fig. 4B, suggest some unique patterns among these silicon sample populations. For example, within the population simulated by Falcon-180B, statements are on average less commonsensical than they are to humans (most scores lie above the diagonal perfect-calibration line). In addition, while they have roughly the same correlation with humans, for Mixtral-8x7B statement commonsensicality scores tend to be collapsed to polar values of 0 and 100%, whereas for Falcon-180B and Flan-T5-XXL they tend to spread more evenly.

Similar to Mixtral-8x7B, the tendency of statement commonsensicality to be close to extreme values is common among many models like Gemini 1.0 and Claude 3 Opus, also shown in Fig. 4B. In other words, statements are either very commonsensical or the opposite according to the ratings by the simulated populations from these models. This is partly a result of LLMs being extremely confident in their answers to our prompts by outputting probabilities close to 0 or 1. (Contrast this with what we observe in humans, depicted in the black histogram to the right end of Fig. 4B.) While the cause of this “uniformity” (58) in their answers is uncertain, it is likely because these models have gone through extensive instruction fine-tuning and human alignment, two phases of training that tend to collapse their outputs toward singular choices associated with high rewards (41, 59, 60)—thereby sacrificing their distributional representativeness.

Finally, we explore the informativeness of *all* models at once by performing a regression analysis predicting a statement’s score in humans using the same 35 scores in all silicon sample populations. As can be seen in Fig. 4C, taking an ensemble of all models does indeed improve the prediction of statement scores; average out-of-sample $R^{2}=0.36$.

In summary, this section views every LLM as an aggregator of human knowledge on a large scale, and tests whether the population simulated by this model is an adequate representation of real humans. Once again, the heterogeneity in human commonsense beliefs is evidenced by a moderate split-half reliability of r=0.60, but the best model-generated silicon samples can only correlate with humans at r=0.43, well below this baseline. In addition, while a statement’s score in a silicon-sample population is predictive of its score in the human population, the majority of the variation in the latter is left unaccounted for.

## Discussion

Designing machines that exhibit human-like tendencies in both individual and social environments is a central objective of AI research (61–63). This often requires AI models to attain some form of commonsense intelligence (64), of which commonsense knowledge is an important constituent (4). While standardized benchmarks are a straightforward method to evaluate progress in this area, we argue that the notion of invariant ground truth—widely assumed but rarely articulated in benchmarking practices—has likely misguided AI researchers, creating an illusion of success as LLMs continue to pass a growing number of tests (47, 65–67).

Our argument is motivated by the heterogeneity in the content of human commonsense knowledge. Since a correct label is unlikely to exist in this domain, what a model “gets right” may be meaningful to some people but not so much to others (68, 69). This “one-truth myth” (70) manifests itself in many domains involving subjective interpretation (71) such as toxicity detection (72), image classification (73), moral judgment (74–76), rhetoric decoding (77), and even medical relations understanding (70). Several works, in response, have called for treating empirically observed human judgments as a normative standard for human-like AI benchmarking (25, 78–81). Following this direction, we propose two interpretations of LLMs—as independent survey participants and as generators a silicon population—and evaluate the degree to which their elicited ratings correspond to the distribution of human opinions observed on a large scale.

The proposed evaluation framework rests on several assumptions and design choices. For example, to obtain an LLM’s opinion distribution, we rely on the probabilities of its generated tokens. Other approaches, such as repeated sampling or verbalization (82), have shown some comparative success but are not employed in this article. Regardless, there is an underlying frequentist assumption that the model’s internal confidence in a statement is to some extent calibrated to the empirical frequency with which humans—especially those who have contributed to its training dataset—would endorse the same statement. This is not theoretically substantiated but is rather an empirical demonstration among recent work that studies how LLMs can be used to approximate public opinion (32, 35, 37, 51). In addition, a model’s calibration to the actual frequency of a belief can be controlled by its sampling temperature. In our case, this parameter is set to 1.0 (see the Methods section) because we are primarily concerned with evaluating the model in its default setting rather than fine-tuning it for out-of-sample prediction.

We also posit that the most significant factor that predisposes an LLM toward a commonsense belief is its training data. However, another driver that may explain the model’s response is its inherent sycophantic tendency: rather than being faithful to the data, it could simply be attempting to look for whatever answer it thinks the user wants. Although it is difficult to isolate either of these two drivers, we can at least inspect what happens if we further clarify to the model the role it is supposed to play. The results presented in SI Appendix, Section E, which include the judgments and reasoning by some GPT models in response to several prompting variants, suggest that these models are likely role-playing independently rather than making an effort to impute what their user may believe. While not attempted in this work, an in-depth analysis of LLM reasoning can also help unpack certain behaviors of these models, such as when they subjectively endorse a statement but predict that most others would not.

Human and model common sense, in addition, depends on what kinds of statements they are asked to rate. In SI Appendix, Section F, we show that the calculation of commonsensicality is sensitive to different features of a statement. For instance, almost all models appear significantly more commonsensical if they only rate statements that depict a fact or describe the physical world. The opposite is observed if these statements use a figure of speech or describe the social world instead. Our corpus of choice, moreover, is likely not representative of the total stock of human commonsense knowledge. Some areas posited to be in this domain—e.g. theory of mind inferences (16, 17)—have received increasing attention from the AI community but are largely missing in our context. Thus, the performance metrics reported here may only reflect certain parts of a much bigger picture of human and machine common sense. Curating a larger statement collection, guided by an established taxonomy (18, 83), spanning different languages, and involving more concrete tasks, will address this potential incomprehensiveness. As we have remarked earlier, however, our model assessment methodology is agnostic to the very content of such statements, and therefore can be readily applied to any new corpus used in future work.

Beyond these considerations, it is worth reiterating that the term “humans” used in this work refers more precisely to the group of English-speaking participants recruited on Amazon Mechanical Turk. In focusing only on their aggregated ratings, our framework does not take into account the potential importance of any cognitive and behavioral diversity among individuals in this sample. Past research, however, has found that social perceptiveness—the ability of a person to “read” other people’s emotions—can reliably predict commonsensicality (24). Thus, future work can consider enhancing the fidelity of LLM simulations by having this variable encoded in a model’s persona when it engages in role-playing (28, 31, 51, 84–86), although this method is not without caveats (87–91). This work additionally leaves an open question of what systematic characteristics of a group and its social processes can determine the content of its shared knowledge. Thus, it is unclear, for example, how the commonsensicality of GPT-4o (which ranks below two-thirds of humans in our MTurk sample) would change if it were considered only alongside American college students. Significant depth can be added to future work by exploring elements of commonsense knowledge that are (near-)unanimously shared, or, when they are only locally shared, how exactly they vary from one group to another (92, 93)—and how the common sense of an AI model is expected to change accordingly.

Throughout history and across discussion spheres, the term “common sense” is notably overloaded (4, 23, 94). Our exposition, in particular, views AI common sense *only* as the ability to synthesize statistical regularities in an LLM’s training data, then assesses if this synthesis can lead to a good approximation of the heterogeneity of human knowledge observed in reality. This approach is from an entirely pattern recognition point of view. By contrast, a primary focus of cognitive psychology is to devise, and replicate in AI, a formal mechanism for human commonsense capabilities (95). This model-building approach, which addresses *why* humans and LLMs respond the way they do to the statements (96), is not attempted in this article. Thus, the foregoing results are necessarily limited to the content of commonsense knowledge, as articulated by the human participants on MTurk, and are not intended to suggest any individual-level cognitive mechanism for commonsense psychology, either in humans or in LLMs.

## Methods

We use the dataset in Ref. (24) which contains N=4,407 statements sourced from seven domains: the news media via Google News (N=290), political campaign emails during US elections (97, N=668), AI corpora like ConceptNet (98, N=581), and ATOMIC (99, N=697), aphorisms taken from books (N=709), and statements elicited by online participants—either via completing a short sentence (N=630) or in response to a prompted domain of knowledge (N=832). See SI Appendix, Table S2 for some examples. Most statements are short with a median of 11 words. Human participants (N=2,046) were recruited from Amazon Mechanical Turk to rate these statements. The human sample contains English-speaking residents in the United States, is 44 years old on average, and has more female (51.2%) than male (48.3%) participants (SI Appendix, Table S1 presents more descriptive statistics). Given each statement, the human workers answered the following questions: (a) “Do you agree with this statement?” and (b) “Do you think most other people would agree with this statement?” The term “other people” in question (b) was not intended to refer to any specific population, so its interpretation was entirely up to the human raters (and LLMs by extension). Each participant was tasked with labeling 50 randomly chosen questions, and on average each statement received ratings from 23 participants. The human data collection protocol was reviewed by the University of Pennsylvania Institutional Review Board (IRB, protocol 849601) which determined that the proposal met eligibility criteria for IRB review exemption. All human subjects provided informed consent before participating.

### Large language models and their ratings

We make use of 35 widely used instruction-finetuned large language models. These include open-weight models (i.e. those whose weights are openly accessible) and closed-source models. The full list can be found in SI Appendix, Table S3. All open-weight models are loaded via the Hugging Face library in Python. For closed-source models, we directly use the APIs provided by their creators.

For every statement, we ask each LLM the same two questions (a) and (b). Each question is asked in a separate chat session, in order to eliminate any influence of the chat history on the answer. We design each prompt so that the model’s answer is expected to start with a definitive “yes” or “no.” Exact prompts and conversation settings are the same for all models and can be found in SI Appendix, Section B.

To generate an answer, the model uses a parametric probability distribution over its vocabulary to sample new tokens, one at a time. We extract the probabilities of the tokens “yes” and “no” and discard all other tokens, then rescale these two probabilities so they add up to one. For all open-weight models, this distribution can be accessed directly. For GPT-3.5/4, we can access the probabilities for up to the top 20 tokens. For Gemini, Claude 3, Mistral-Large, and GPT-5, we perform repeated sampling, where we ask a model the same questions multiple times and report the empirical frequencies of their generated answers. See SI Appendix, Section B.

The probability that the model answers “yes” to question (a) is called its rating distribution; see SI Appendix, Eq. 12. This can be considered as the model’s inherent confidence in agreeing with a statement. Figures S4 and S5 examine the calibration between this confidence and the relative frequency with which humans would give the same response for some models. The output probability is also crucially dependent on the sampling temperature. It is possible that the model can be more calibrated using a different temperature, but our goal is to evaluate it in its default mode, corresponding to using the temperature of 1.0. When binary decisions are called for, we choose the answer—“yes” or “no”—that is associated with the higher probability, also called the “argmax” of the model’s rating distribution. These prompting settings were preregistered prior to data collection on AsPredicted, project number 162475 (100).

### Measuring LLMs’ individual commonsensicality

We adopt the intuition in Ref. (24) and calculate an LLM’s degree of common sense as if it were a real human participant (see Fig. 2A). In particular, to have common sense, the model must agree with the human majority opinion, and correctly predict this consensus irrespective of its subjective opinion. These two criteria are reflected in the model’s answer to questions (a) and (b) above, respectively.

Precisely, for every statement let the human majority rating (agree or disagree) be the opinion held by at least half of the participants. If the model’s answer to question (a) (whether it agrees with the statement) coincides with this majority rating, this counts as a correct answer. The accuracy of the model, averaged over all 4,407 statements, is called its *consensus* score, which depicts how often its subjective rating agrees with the human majority. Similarly, the same accuracy with respect to the model’s answer to question (b) (whether it thinks most people would agree with this statement) is called its *awareness* score, representing how often it predicts the position of the human majority, notwithstanding how it subjectively rates a statement previously via question (a). We combine a model’s consensus and awareness scores by taking their geometric average into its *commonsensicality* score. See SI Appendix, Eqs. 16–18. These three scores, each ranging between 0 and 100%, are presented in Fig. 2B above.

To analyze the relationship between a model’s size and its commonsensicality score, we choose 23 models from 6 model families in our collection: Falcon, Flan-T5, Gemma, LLaMA-2/3, Mistral, and Qwen2. These are families that contain at least two models whose sizes are available to us. The models are presented in Fig. 2C. We then perform a linear mixed-effects regression analysis with commonsensicality as the outcome, model size as the fixed effect and model family as the random effect. A detailed setting can be found in SI Appendix, Eq. 19.

To investigate the relationship between an LLM’s general performance on a popular benchmark and its commonsensicality, we extract the LMArena Elo scores (46), last updated on 2025 August 16. Essentially, LMArena tests a total of 230 LLMs side by side and uses crowd-sourced human ratings to rank these models. A participating user interacts with two randomly chosen LLMs at the same time, asking them the same question and receiving answers from both. Then, the user indicates which model is better. From ∼3.87 million such comparisons, models are assigned an Elo score that depicts their relative performance. More detail can be found in SI Appendix, Section C, especially SI Appendix, Eq. 20. We are able to find Elo scores for 24 models, and in Fig. 2D, we report the Pearson correlation between a model’s Elo score and its commonsensicality.

Since the calculation of model commonsensicality is exactly how human participants are measured on their common sense—see SI Appendix, Eqs. 9–11—this allows us to compare every LLM against every human directly. One major difference is that while every model can label all 4,407 statements, every person was only asked to label a random subset of 50 statements. To make a fair comparison, for each LLM–human pair, we restrict the calculation of their commonsensicality scores to the 50 questions that the participant previously rated. More detail can be found in SI Appendix, Section C. Figure 2E depicts how often a model is judged to be more commonsensical than a human.

### Analyzing LLMs as simulators of silicon sample populations

Recall that when we ask an LLM questions (a) and (b) above, we record the probability with which it answers “yes” or “no.” In the limit, this probability can be interpreted as the frequency of answers by the silicon samples simulated by this model. For example, if an LLM answers “yes” with 70% probability, then among 1,000 silicon samples that are generated by this model, 700 of them are expected to say “yes.”

For each population (humans or silicon samples), we analyze common sense on the statement level. Here, we describe this calculation with respect to the human population, more details of which can be found in SI Appendix, Section A. Let $d_{i}^{h,a}$, called the human rating distribution, be the proportion of people who indicated they agreed with statement *i*, via question (a). The majority opinion for this statement is then ${majority}_{i}^{h}=1[d_{i}^{h,a}\geq 0.5]$, which is 1 if at least half of the people agreed with it, and 0 otherwise. The consensus score measures how far people are from a unanimous opinion: $c_{i}^{h}=2\times |d_{i}^{h,a}-0.5|$. It is 0 when exactly half of the people agree with it, and 1 when everyone agrees or disagrees with it. The awareness score measures how accurately people predicted the majority rating via question (b). If the ${majority}_{i}^{h}=1$, then the statement’s awareness, $a_{i}^{h}$, is the proportion of people who predicted most others would agree; otherwise, it is the proportion who predicted most others would disagree. The commonsensicality score is defined as $m_{i}^{h}=\sqrt{c_{i}^{h}\times a_{i}^{h}}$. We denote the same scores for the model-simulated population with the superscript *m* instead of *h*, such as $m_{i}^{m}$.

For example, in Fig. 3A, we observe that 86% of humans agree with the statement, “Experience is imperative to run a country,” while 95% of the same people thought most others would agree with it. Hence, $d_{i}^{h,a}=0.86$, which means the majority rating is 1, or “agree.” The consensus score is thus $c_{i}^{h}=2\times |0.86-0.5|=0.73$, and the awareness score is $a_{i}^{h}=0.95$, because 95% of the people correctly predicted that it would receive an “agree.” The commonsensicality score is, therefore, $m_{i}^{h}=\sqrt{0.73\times 0.95}=0.83$. The same scores ($c_{i}^{m}$, $a_{i}^{m}$, and $m_{i}^{m}$) are identically defined for the population of LLM-based silicon samples.

Each statement in our corpus was labeled using six epistemological dichotomies. Online participants were asked to rate whether a statement depicts a *fact* (something that can be objectively demonstrated to be true) vs. an *opinion* (something that might be held true by some but not by others); describes *physical* reality (objective features of the world according to, say, physics and biology) vs. *social* beliefs, perceptions, and rules that govern human experience; uses *literal language* that means exactly what it says vs. a *figure of speech* such as an aphorism or metaphor; conveys *positive* empirical regularities in the world (e.g. “Hot things will burn you.”) vs. a *normative* judgment, belief, value, or convention (e.g. “Treat others how you want them to treat you.”); conveys declarative *knowledge* about the world vs. a *reasoning* that involves both knowledge and logic; and depicts *everyday* experiences of humans vs. *abstract* rules and regularities that must be synthesized from experience. See SI Appendix, Section D for more detail.

In Fig. 3C, we calculate the reported differences as follows. First, we separate the entire corpus of N=4,407 into two categories: those that depict a fact and those that portray a personal opinion. Each statement, again, has a commonsensicality score $m_{i}^{h}$ (assuming we are considering the human population). We calculate the average difference in this score between the fact-like statements and the opinion-like statements. To quantify uncertainty, we perform this calculation via 1,000 random bootstraps. The mean difference and the 95% bootstrapped CI are depicted in the figure. This applies to other dichotomies as well as the populations of silicon samples by LLMs.

In Fig. 4A, we compute the Pearson correlation between $m_{i}^{h}$ and $m_{i}^{m}$—the same commonsensicality scores for every statement within two populations: humans vs. silicon samples. All two-sided *P*-values are Bonferroni-corrected. In Fig. 4B, we show this result in more detail via scatter plots for five models: Falcon-180B, Flan-T5-XXL, Mixtral-8x7B, Gemini Pro 1.0, and Claude 3 Opus. We also fit a linear model, predicting $m_{i}^{h}$ using $m_{i}^{m}$, and plot the best-fit line within these plots. The out-of-sample coefficient of determination, $R^{2}$, for this linear model is also depicted. Finally, in Fig. 4C, we combine all 35 statement scores $m_{i}^{m}$ in 35 silicon sample populations to predict the same score in humans, $m_{i}^{h}$.

## Supplementary Material

pgag029_Supplementary_Data

## Data Availability

All data and code to reproduce the results in this article have been deposited to Zenodo at https://doi.org/10.5281/zenodo.17281355 and can also be found on GitHub at https://github.com/Watts-Lab/commonsense-llm-eval.

## Notes

a In Fig. S7, we illustrate this point further by showing in greater detail the difference between $m_{i}^{m}$ and $m_{i}^{h}$. In particular, we calculate the mean absolute error (MAE) between these two scores, which represents the average difference in statement commonsensicality between humans and silicon samples. The highest-ranked model is Flan-T5-XXL, with the smallest MAE of 21.41. This means that on average, the commonsensicality score of a statement, according to Flan-T5-XXL’s silicon samples, is 21.41 points away from the score of the same statement according to humans. This error is the highest for Falcon-40B, with an MAE of 52.69.

## Preprints

This manuscript was posted as a preprint at https://arxiv.org/abs/2505.10309.
