# Supplementary material for: A large-scale evaluation of commonsense knowledge in humans and large language models
Source: PNAS Nexus. 2026 Feb 16;5(3):pgag029. doi: 10.1093/pnasnexus/pgag029 (PMC12947942; doi:10.1093/pnasnexus/pgag029)
Supplement: pgag029_Supplementary_Data [file pgag029_supplementary_data.pdf]

Supplementary Information for
A large-scale evaluation of commonsense knowledge in humans and large language models

Tuan Dung Nguyen[a,1], Duncan J. Watts[a,b,c], and Mark E. Whiting[a,b,d]

[a] Department of Computer and Information Science, University of Pennsylvania, Philadelphia, PA 19104, USA.
[b] Operations, Information and Decisions Department, The Wharton School, University of Pennsylvania, Philadelphia, PA 19104, USA.
[c] Annenberg School for Communication, University of Pennsylvania, Philadelphia, PA 19104, USA.
[d] Pareto, San Francisco, CA 94111, USA.
[1] Corresponding Author: Tuan Dung Nguyen. Email: joshtn@seas.upenn.edu.

Table of Contents

A Measuring Common Sense in Humans and Statements **2**
- A.1 Statement-Level Commonsensicality . . . 5
- A.2 Human-Level Commonsensicality . . . 6

B Collecting Responses from Large Language Models **8**
- B.1 Designing Prompts and Expected Responses . . . 8
- B.2 Extracting Responses from LLMs . . . 11

C Measuring Individual-Level Commonsensicality in LLMs **16**
- C.1 Model Size and Commonsensicality . . . 16
- C.2 Model General Performance on LMArena and Commonsensicality . . . 17
- C.3 Model Performance on Static Commonsense Benchmark and Commonsensicality . . . 17
- C.4 Comparing Models and Humans Side by Side on Commonsensicality . . . 22
- C.5 Alternative Calculation of Model Commonsensicality with Model-Contributed Majority Vote . . . 22

D Measuring Commonsensicality when LLMs are Simulators of Silicon Samples **23**
- D.1 Comparing Statement Commonsensicality Scores within Humans and Silicon Samples by Statement Type . . . 23

E Other Variants of Model Evaluation Settings **27**
- E.1 Model's Reasoning Following Binary Answer . . . 27
- E.2 Model's Reasoning Before Binary Answer . . . 27
- E.3 Clarifying the Role of LLMs in the Survey . . . 27

F Analyzing Commonsensicality for Various Types of Statements **34**

A Measuring Common Sense in Humans and Statements

Here we describe the commonsensicality framework by Whiting and Watts (*1*), whose data we adopt to study common sense in LLMs.

From September 2 to September 14, 2022, a total of 2,141 individuals were recruited on Amazon Mechanical Turk to perform statement rating. All but 10 participants were from the United States. The exceptions were 3 individuals from Canada, and one individual from each of the following countries: Bangladesh, Romania, France, Mexico, Italy, Germany and Australia.

Only individuals who self-declared—via explicit answers throughout the session—to give honest, accurate and non-random answers were included. In addition, only individuals who participated in both the Reading the Mind in the Eyes Test (*2*) and the Cognitive Reflection Test (*3*) were included. This gives a final sample of 2,046 individuals. Some descriptive statistics of this sample are given in Table S1. More information about the survey setup can also be found in the Supporting Information of ref. *1*.

Suppose we have a set of $n_s \geq 1$ statements (see Table S2 for an example) and a set of $n_p \geq 1$ people. (In this case, $n_s = 4,407$ and $n_p = 2,046$.) Given a statement, such as "If you want to play a *[sic]* guitar then you should take lessons," a human participant was asked two questions:

(a) "Do you agree with this statement?"

(b) "Do you think most people would agree with this statement?"

Common sense is measured for both humans and statements. The underlying rationale is that a person with common sense knows what is true (in the sense that their belief coincides with the general human consensus), and also knows what other people believe to be true (in the sense that they can accurately predict what the majority of other people believe). Similarly, a statement is common sense if it is (nearly) unanimously agreed upon by people in this population, who must also accurately predict what the human consensus on this statement is. All of this depends on participants' answers to the two questions above.

To make this precise, define A be the $n_s \times n_p$ matrix such that

$$A_{i,j} = \begin{cases} 1 & \text{if the } j\text{-th participant answered "yes" to question (a) about the } i\text{-th claim} \\ 0 & \text{if the } j\text{-th participant answered "no" to question (a) about the } i\text{-th claim.} \end{cases} \tag{1}$$

In other words, the matrix A contains participants' answers to question (a) above. Similarly, let B be the $n_s \times n_p$ matrix containing answers to question (b) such that

$$B_{i,j} = \begin{cases} 1 & \text{if the } j\text{-th participant answered "yes" to question (b) about the } i\text{-th claim} \\ 0 & \text{if the } j\text{-th participant answered "no" to question (b) about the } i\text{-th claim.} \end{cases} \tag{2}$$

One complication in this study is that not every claim was labeled by everyone, because every participant was required to label only a small number of claims, 50 to be exact. Every (i, j) entry in matrices A and B, therefore, is only valid if the i-th claim was indeed assigned to the i-th participant; otherwise, participant j would have never seen and labeled claim i in the first place, and hence the entry must not be taken into account. Hence, A and B are partially filled matrices.

Define $\Omega_i \subseteq \{1, 2, \ldots, n_p\}$ as the set of participants who were indeed assigned to rate the i-th statement, and $\Phi_j \subseteq \{1, 2, \ldots, n_s\}$ as the set of statements that were indeed assigned to the j-th participant. We hence have $|\Phi_j| = 50$, where $|\cdot|$ denotes the size of a finite set.

Before we go on to define commonsensicality, define the *human rating distribution* for each statement i as the proportion of people that indicated they agreed with statement i (*i.e.*, by answering "yes" to question (a)):

$$d_i^{h,a} = \frac{1}{|\Omega_i|} \sum_{j \in \Omega_i} A_{i,j}. \tag{3}$$

Here, the subscript i denotes the index of a statement; the superscripts h and a indicate that this is the distribution based on human ratings, and with respect to question (a), respectively. For example, if 70% of people who were

Type	Level	Share of level (%)
Age	21–39	42.52
M = 44.25, SD = 12.87	40–59	42.03
Median = 42.00	60–79	15.34
Min = 21, Max = 85	80+	0.10
Gender	Female	51.22
	Male	48.29
	Other	0.49
Race	White	80.50
	Black	7.87
	Asian	8.21
	Indigenous	1.52
	Other	1.91
Education	Less than high school	0.59
	High school diploma	9.58
	Some college or vocational degree	17.64
	2-year college degree	10.22
	4-year college degree	43.84
	Post-college degree	13.83
	Professional degree (e.g, J.D., M.D.)	2.44
	Doctoral degree	1.86
Political party	Democrat	50.24
	Republican	26.00
	Independent	23.75
Politics: social views	Strongly liberal	25.51
	Somewhat liberal	32.01
	Moderate	16.13
	Somewhat conservatie	16.28
	Strongly conservative	10.07
Politics: fiscal views	Strongly liberal	17.89
	Somewhat liberal	28.40
	Moderate	18.91
	Somewhat conservatie	23.07
	Strongly conservative	11.73
Marital status	Single never married	36.61
	Married or domestic partnership	51.76
	Separated	0.83
	Divorced	9.19
	Widowed	1.61
Income	Less than $10,000	3.52
	$10,000 to $19,999	6.45
	$20,000 to $29,999	9.87
	$30,000 to $39,999	10.46
	$40,000 to $49,999	10.95
	$50,000 to $59,999	11.97
	$60,000 to $69,999	7.04
	$70,000 to $79,999	8.60
	$80,000 to $89,999	5.18
	$90,000 to $99,999	6.40
	$100,000 to $149,999	13.10
	$150,000 or more	6.45

Table S1. Descriptive statistics of the human sample (N = 2,046).

Data source	*N*	Examples
News media	290	- A low covid positivity rate should allow for restrictions to be loosened.<br>- Children should not be taught to be ashamed of their own skin color.<br>- Providing access to loans for producers boosts agricultural economy during crisis.<br>- We should aim to put our responsibilities before any personal or political agenda.<br>- The proliferation of charter schools affects the ability of states to efficiently distribute tax dollars.
Campaign emails	668	- Abolish the police is an extreme stance taken by democrats.<br>- Access to healthcare should be available for all without worry of debt.<br>- Partisan politics and the democrats have held up real change.<br>- The health care system can help a lot of americans that need jobs.<br>- We should support freedom transparency and accountability in our elections.
ConceptNet	581	- A ball is round.<br>- A desire to share knowledge would make you want to teach other people.<br>- If you want to compete against someone then you should enter a competition.<br>- Something that might happen as a consequence of having a conversation is exchange of information.<br>- The first thing you do when you surf the web is turn on your computer.
ATOMIC	697	- If max accepts sam invitation then max intends to to gain person's friendship.<br>- If max [*sic*] about to get married then max wants to be happily married.<br>- If max accepts the job then max intends to to be employed.<br>- If max accompanies sam far then max intends to to keep sam company.<br>- If max attracts sam's attention then sam feels curious about max.
Aphorisms	709	- A doctor and a farmer know more than a doctor alone.<br>- It is always the best policy to speak the truth unless of course you are an exceptionally good liar.<br>- Let us never negotiate out of fear but let us never fear to negotiate.<br>- Live your life as though your every act were to become a universal law.<br>- Without craftsmanship inspiration is a mere reed shaken in the wind.
Situational response	630	- If a person digs themselves into a hole they will be in a bad mood.<br>- If alex calls sam's mother they would have a conversation about sam.<br>- If alex lands a job they will arrive early the first day of work to make a good impression.<br>- If alex sees a bat he will poke the bat.<br>- If alex works the night shift they will sleep during the day so they are awake for work.
Categorical response	832	- 5 is a lot bigger than 1.<br>- Advancements in technology can help save lives and improve the quality of life for everyone in the world.<br>- Computers can never be an actual human.<br>- If john wants to be a teacher he would apply for a teaching position.<br>- Pausing before proceeding can lead to better results.

Table S2. Seven sources of statements in our corpus, taken from Whiting and Watts (*1*). For ATOMIC (*4*), people's names such as "max" and "sam" were used to replace entities such as "PersonX" and "PersonY."

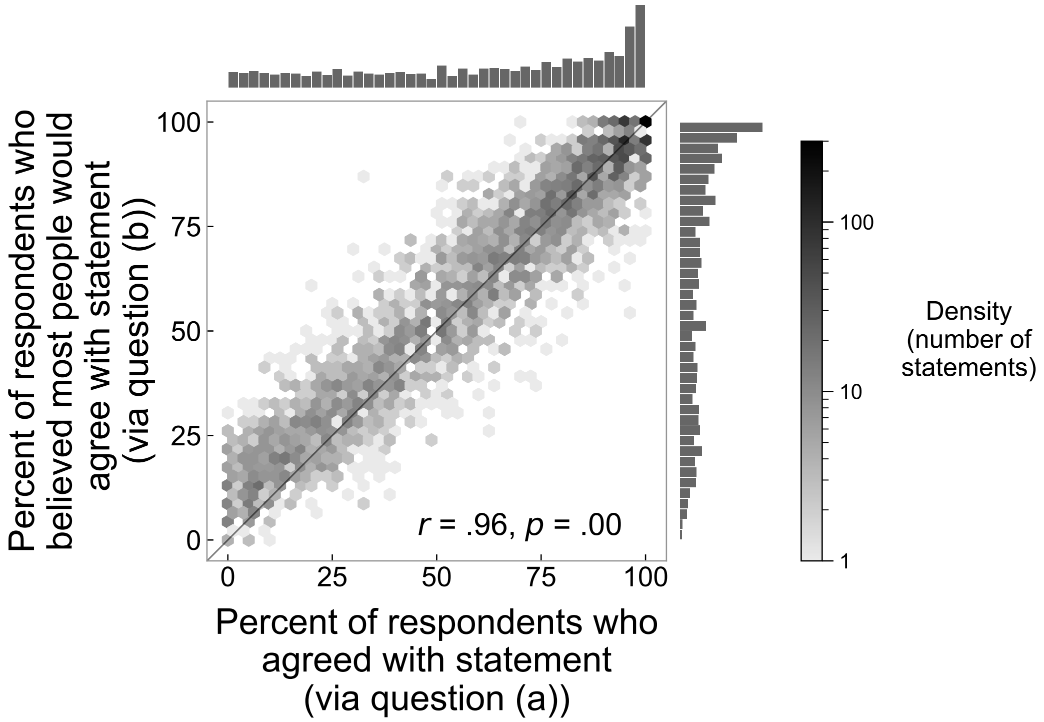

Figure S1. Summary of the human ratings collected by Whiting and Watts (*1*). Participants (N = 2,046) were given a statement (N = 4,407) and asked to indicate (a) whether they agreed with the statement and (b) whether they thought most people would agree with the statement. Each participant was assigned only 50 randomly chosen statements, and on average, each statement received 23 unique ratings. Every statement is depicted in this figure, where the x-axis represents the percentage of people who agreed with it, $d_i^{h,a}$ in Equation 3, and the y-axis represents the percentage of people who believed most others would agree with it, $d_i^{h,b}$ in Equation 5.

assigned to rate statement i agreed with it, then $d_i^{h,a} = 0.7$. If we take the mode of this distribution, the *human majority rating* of each statement i is

$$\texttt{majority}_i^h = \mathbb{1}\left[d_i^{h,a} \geq 0.5\right], \tag{4}$$

where the indicator function $\mathbb{1}[\cdot]$ is equal to 1 if the given argument is true and 0 otherwise. Unlike the human rating distribution, the human majority rating is a binary variable and is equal to 1 if and only if at least half of the participants who rated i agreed with it. Note that we resolve ties, where exactly half of the participants agreed with a statement, with a final label of 1, or "agree." Note also that we only count ratings by participants in Ω_i because they were the only ones assigned to rate statement i.

Similarly, the proportion of human participants who think that most other people would agree with statement i (*i.e.*, by answering "yes" to question (b)) is

$$d_i^{h,b} = \frac{1}{|\Omega_i|} \sum_{j \in \Omega_i} B_{i,j}. \tag{5}$$

Figure S1 on page 5 shows the proportions of human participants who responded "yes" to questions (a) and (b) for all statements.

A.1 Statement-Level Commonsensicality

The commonsensicality of a statement is a combination of its two scores, consensus and awareness.

Given a statement i, its *consensus* score measures how close people's judgments of this statement are to absolute

unanimity:

$$c_i^h = 2 \times \left| d_i^{h,a} - 0.5 \right|. \tag{6}$$

High consensus requires that the human rating distribution $d_i^{h,a}$ be very close to 0 (when everyone disagrees with i) or 1 (when everyone agrees with i). Note that this definition is symmetric with respect to the polarity of opinions. For example, a statement that was agreed upon by 60% of participants would have the same consensus score as a statement that was disagreed upon by 60% of participants.

In addition, given statement i, its *awareness* score measures how accurately people perceived its majority rating. Formally, we have

$$a_i^h = \frac{1}{|\Omega_i|} \sum_{j \in \Omega_i} \mathbb{1} \left[B_{i,j} = \texttt{majority}_i^h \right]. \tag{7}$$

High awareness requires that people know what most others believe, which is recorded in question (b) above.

For a statement to be common sense, it must score high in both consensus and awareness. The *commonsensicality* score of statement i is defined as the geometric mean of its consensus and awareness scores:

$$m_i^h = \sqrt{c_i^h \times a_i^h}. \tag{8}$$

The commonsensicality score m_i^h is also between 0 and 1, and values closer to 1 indicate higher degrees of common sense.

Figure S2 (bottom) on page 7 presents statements on two dimensions: consensus and awareness. Generally, statements received higher awareness scores than consensus scores. The figure also shows that most statements received a high commonsensicality score, with a median of 70.12%.

A.2 Human-Level Commonsensicality

Commonsensicality is also defined for people, comprising consensus and awareness scores.

Given a person j, their *consensus* score is defined as the fraction of times they agreed with the majority rating across all statements they rated:

$$C_j = \frac{1}{|\Phi_j|} \sum_{i \in \Phi_j} \mathbb{1} \left[A_{i,j} = \texttt{majority}_i^h \right]. \tag{9}$$

To have high consensus, person j's own ratings of the statements, via their answer to question (a) above, must coincide with the overall human majority ratings.

The *awareness* score of person j is the fraction of time they accurately predicted the majority rating across all statements:

$$A_j = \frac{1}{|\Phi_j|} \sum_{i \in \Phi_j} \mathbb{1} \left[B_{i,j} = \texttt{majority}_i^h \right]. \tag{10}$$

To have a high awareness score, person j's prediction of what most other people think—via question (b) above, and regardless of what they chose for question (a)—must also coincide with the overall human majority ratings.

Consensus C_j and awareness A_j scores are also between 0 and 1. Similarly, we take the geometric mean of these scores to give participant j's *commonsensicality* score as

$$M_j = \sqrt{C_j \times A_j}. \tag{11}$$

This score is also between 0 and 1, with higher values indicating higher degrees of common sense in a person.

Figure S2 (top) also presents humans on two dimensions: consensus and awareness. Different from statements, humans tended to exhibit very similar scores on both dimensions (most of them lie close to the diagonal line). The median commonsensicality for humans is 79.43%.

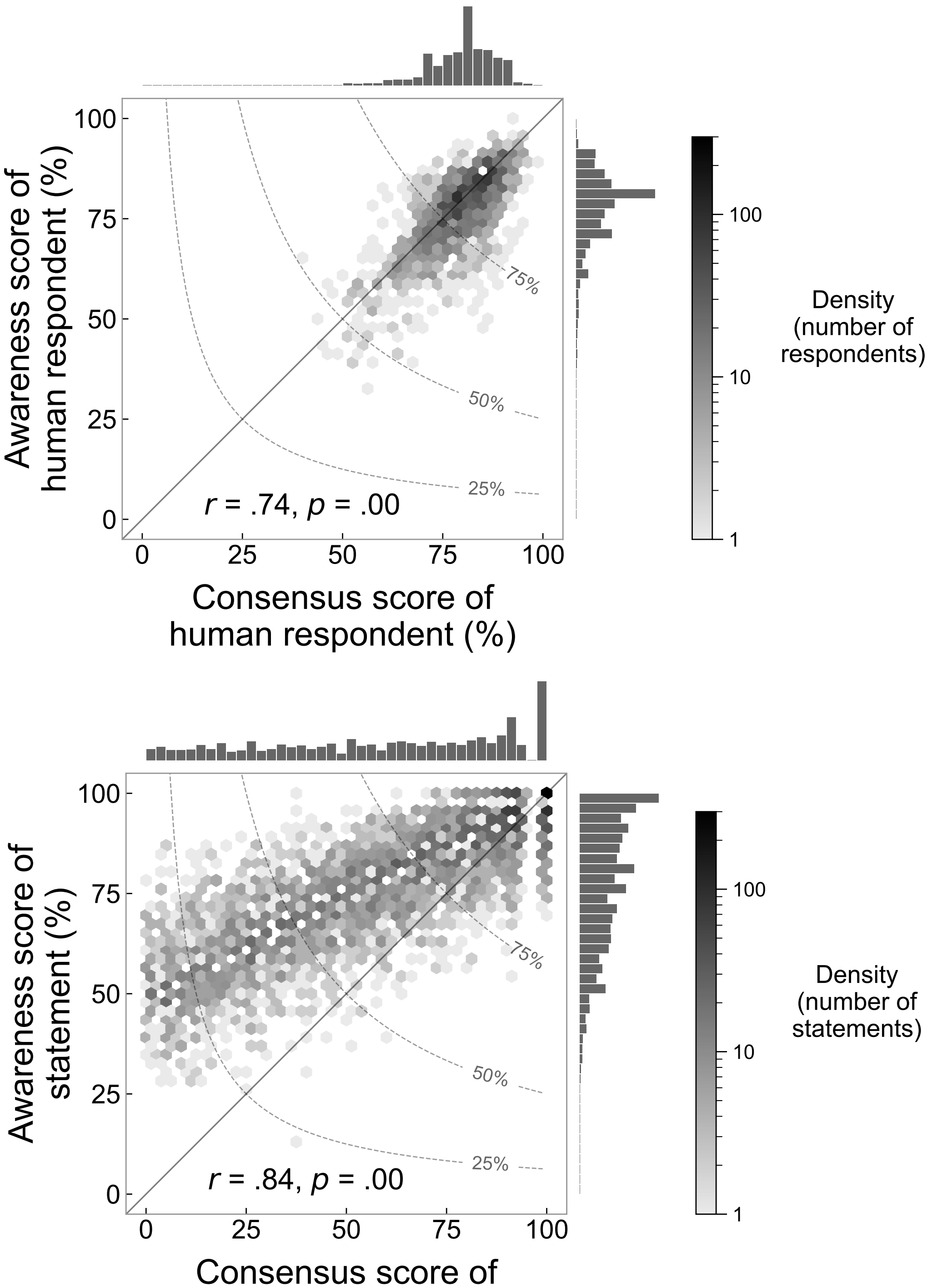

Figure S2. Top: Individual-level commonsensicality, M_j in Equation 11, defined for every human participant. Bottom: collective-level commonsensicality, m_i^h in Equation 8 defined for every statement. The commonsensicality score is the geometric average of the consensus and awareness scores, which are depicted in these figures. The three level curves depict combinations of consensus and awareness that produce three levels of commonsensicality: 25%, 50%, and 75%.

B Collecting Responses from Large Language Models

To assess common sense in LLMs, we select a number of models that have been trained to follow conversations (instruction-tuned models). The full list of models we experiment with is found in Table S3 on page 9. A typical conversation with an LLM is as follows.

```
[SYSTEM PROMPT]
[USER PROMPT]
[ASSISTANT RESPONSE]
[USER PROMPT]
[ASSISTANT RESPONSE]
...
```

The conversation starts with an optional *system prompt*, which is used to steer the general behavior of the LLM. For example, one can set this to "You are a debate expert" if the model is intended to be used to make arguments. Then, the system prompt is followed by a series of alternating *user prompts* and *assistant responses*. All messages in this conversation are concatenated into a *chat history*, which is a string of the form `[SYSTEM PROMPT] [SEP] [USER PROMPT] [SEP] [ASSISTANT RESPONSE] [SEP] [USER PROMPT] [SEP] [ASSISTANT RESPONSE]...` The `[SEP]` symbol is a special token denoting the separation of messages, and is specific to each model.

To use an LLM to generate an assistant response, one prepares a chat history that ends with a user prompt, and hence expects an assistant response to follow from it. The model takes the chat history as input and autoregressively generates new tokens one by one, until a finishing condition is reached. These tokens are then appended to the chat history, thereby completing the assistant response. Instruction-tuned models are specifically trained in this manner, via many such conversations, and hence we choose these models for assessment.

Code Block S1 shows a concrete example of our procedure, which is described in more detail in the subsections below.

B.1 Designing Prompts and Expected Responses

We decide not to use any specific system prompt. For some models, this is set to their default system prompts, such as "You are a helpful assistant." For some other models, this can be left blank, so the chat history actually starts with a user prompt.

For each of the $n_s = 4,407$ statements, we prompt a model to respond to the following two use prompts:

(a) `Consider the following statement, "[STATEMENT]" Do you agree with this statement? Start your answer with a "yes" or "no".`

(b) `Consider the following statement, "[STATEMENT]" Do you think most people would agree with this statement? Start your answer with a "yes" or "no".`

These are the same as questions (a) and (b) described in Section A. We note three details of these prompts. First, questions (a) and (b) are used in two separate chat sessions. This is because any chat history can modify a model's answer in unexpected ways, which we do not like to experience. Second, we include the sentence "Start your answer with a 'yes' or 'no'." at the end of each prompt to instruct the model to output a "yes" or "no" directly. Without this explicit instruction, the model may start its response with, for example, "I agree with the answer," or give some reasoning before giving its final answer. Either way, extracting the binary answer would be more complicated and noisy. And third, in framing the questions in this way, we need the model to output only one token—"yes" or "no"—and we can extract the probability with which it generates either token, which represents the model's inherent uncertainty in its answer (more below).

Model family	Model name	Model size	Access	Source	Released	Comments
Claude	Claude 3 Haiku	–	Closed	(*5*)	03/24	Most capable
	Claude 3 Sonnet	–	Closed			Medium
	Claude 3 Opus	–	Closed			Least capable
DBRX	dbrx-instruct	132B	Open	(*6*)	03/24	
Falcon	Falcon-7b-Instruct	7B	Open	(*7*)	06/23	
	Falcon-40b-Instruct	40B	Open			
	Falcon-180B-Chat	180B	Open		09/23	
Flan-T5	Flan-T5-Small	80M	Open	(*8*)	10/22	
	Flan-T5-Base	250M	Open			
	Flan-T5-Large	780M	Open			
	Flan-T5-XL	3B	Open			
	Flan-T5-XXL	11B	Open			
Gemini	Gemma-2b-it	2B	Open	(*9*)	02/24	
	Gemma-7b-it	7B	Open			
	Gemini Pro 1.0	–	Closed	(*10*)		Original release
GPT	GPT-3.5-Turbo-0125	175B	Closed	(*11*)	01/24	Original model 2020
	GPT-4-Turbo-0125	–	Closed	(*12*)	01/24	Original model 2023
	GPT-4-Turbo-0409	–	Closed		04/24	
	GPT-4o	–	Closed	(*13*)	05/24	
	GPT-5	–	Closed	(*14*)	08/25	Reasoning model
LLaMA	LLaMA-2-7B-Chat	7B	Open	(*15*)	07/23	
	LLaMA-2-13B-Chat	13B	Open			
	LLaMA-2-70B-Chat	70B	Open			
	LLaMA-3-8B-Instruct	7B	Open	(*16*)	04/24	
	LLaMA-3-70B-Instruct	70B	Open			
Mistral	Mistral-7B-Instruct	7B	Open	(*17*)	09/23	
	Mixtral-8x7B-Instruct	47B	Open	(*18*)	11/23	
	Mixtral-8x22B-Instruct	141B	Open	(*19*)	04/24	
	Mistral-Large	–	Closed	(*20*)	02/24	
OLMo	OLMo-7B-Instruct	7B	Open	(*21*)	02/24	
Qwen	Qwen2-0.5B-Instruct	0.5B	Open	(*22*)	06/24	
	Qwen2-1.5B-Instruct	1.5B	Open			
	Qwen2-7B-Instruct	7B	Open			
	Qwen2-57B-Instruct	57B	Open			
	Qwen2-72B-Instruct	72B	Open			

Table S3. Language models used for our evaluation of commonsensicality. All closed-source models (Claude 3, GPT-3.5/4/5 and Mistral-Large) are accessed via their developers' APIs. All open-weight models are accessed via Hugging Face in Python. For closed-source models, the "–" sign denotes their unknown sizes (number of parameters).

```python
1 # Load tokenizer and model
2 tokenizer = AutoTokenizer.from_pretrained(MODEL_NAME)
3 model = model = AutoModelForCausalLM.from_pretrained(
4     "databricks/dbrx-instruct",
5     device_map="auto",
6     trust_remote_code=True,
7     torch_dtype=torch.bfloat16,
8 )
9 model.eval()
10 model.tie_weights()
11 
12 # These are all the IDs of the tokens in DBRX's vocabulary that map to
13 # the answers "yes" and "no".
14 answer2tokid = {
15     "yes": [9642, 60844, 14331, 58841, 20137, 60665, 7566, 85502, 77830, 95934, 10035, 86508,
       98171, 41898, 14410, 9891],
16     "no": [72719, 6673, 42257, 86176, 43983, 67579, 2822, 79027, 18847, 40305, 34200, 58749,
     5782, 38089, 39522, 73204, 9173, 99076, 2360, 38557, 31415, 12674, 85298, 29466, 2201,
     912, 61559, 17184, 51899, 9278]
17 }
18 
19 # Formulate the prompt
20 messages = [
21     {"role": "user",
22      "content": 'Consider the statement, "A desire to share knowledge would make you want to
     teach other people." Do you agree with this statement? Start your answer with a "yes" or
     "no".'}
23 ]
24 
25 # Encode the message
26 inputs = tokenizer.apply_chat_template(messages, add_generation_prompt=True)
27 
28 # Feedforward the input
29 inputs = torch.tensor(inputs, device=model.device, dtype=torch.long).reshape(1, -1)
30 outputs = model(inputs, output_hidden_states=False)
31 
32 # Extract the probability of immediate next token
33 probs = torch.softmax(outputs[0][0, -1], axis=0)
34 probs = probs.cpu().detach().float().numpy()
35 
36 # Map token IDs to answers
37 answer_probs = {}
38 # The probability that the model answers "yes" is the sum of the
39 # probabilities of all tokens that map to "yes".
40 answer_probs["yes"] = float(probs[answer2tokid["yes"]].sum())
41 # Similar for "no".
42 answer_probs["no"] = float(probs[answer2tokid["no"]].sum())
43 # The probability that the model answers with something else.
44 answer_probs["other"] = 1 - answer_probs["yes"] - answer_probs["no"]
```

Code Block S1. Python code used to extract an open-source model's answer to an example prompt. Here, we demonstrate this process using the model DBRX. Note that there could be multiple tokens within this model's vocabulary that can be mapped to the answers "yes" and "no," hence we need to enumerate them in the `answer2tokid` dictionary on lines 14–17. Based on the prompt (lines 20–23), the model is supposed to start its response with a definitive "yes" or "no." Therefore, we perform one feedforward operation to get the probabilities of all tokens that can be generated next (lines 28–34). We then sum the probabilities of all tokens that map to "yes" and "no" to get the model's probability of answering with either option (lines 36–44).

B.2 Extracting Responses from LLMs

Questions (a) and (b) in Section B.1 are formulated as user prompts for each model, which is then expected to generate a response starting with a "yes" or "no." As explained above, the model does so by autoregression: it maintains a multinomial probability distribution over all possible tokens and randomly chooses a token to generate via sampling from this distribution. In other words, the tokens "yes" and "no" are each assigned a probability with which they are generated. This also means that every other token may be generated, as long as its probability is not zero, leading to "illegal" answers. We address this problem in the following.

First, a review of how we can obtain token probabilities for each model.

- For GPT-3.5 and GPT-4, OpenAI's API allows us to have access to up to 20 tokens associated with the highest sampling probabilities via the `logprobs` parameter.
- For Gemini 1.0 Pro, Claude 3 and Mistral-Large, their APIs do not allow access to these probabilities. We repeatedly ask the same question multiple times and report the empirical frequencies of the generated tokens, which give us an unbiased estimate of the probabilities. We perform 23 repetitions for Claude 3 and Mistral-Large, which matches the average number of people that rated each question in the original dataset (*1*). For Gemini 1.0 Pro, we perform 50 repetitions.
- For GPT-5, we also perform repeated sampling. However, this is a reasoning model which tends to output its "thinking" before giving us its answer, and this tendency cannot be turned off. Thus, in the user prompt, we add the following message—and thus slightly deviate from our preregistration—to discourage this thinking behavior: "Do not include anything else, such as an explanation or reasoning." Each API call allows for 8 repetitions (or "choices"), so for each statement we make 6 API calls, resulting in 48 ratings per statement in total.
- For all other models, which are open-weight and loaded via the Hugging Face library in Python, we have access to the probabilities of all tokens.

In generating tokens, we set all sampling parameters to default; most notably, we keep the sampling temperature at 1.0. Once we have the probabilities of generated tokens, we perform the following processing steps.

- We discard all tokens that do not represent "yes" or "no" answers. This usually happens when a model wants to avoid giving an answer, for example, "As an AI model I cannot…" which leads to the first generated token being "As," an invalid token.
- For some models, their tokenizers have different variants of the same answer, due to formatting reasons. For example, the answer "yes" can be generated from the tokens `Yes`, `yes`, `␣yes`, `"Yes`, *etc.*, which should all be valid. In this case, we compile a list of all such valid tokens and sum over their probabilities to get the final score.
- After this, we have probabilities for the "yes" and "no" answers. Since we have discarded all illegal tokens, these two probabilities do not necessarily add up to one. We hence rescale them by dividing each by their sum. See Code Block S1.

The resulting binomial distribution of answers is called the *model rating distribution* and denoted

$$d_i^{m,a} = p(\text{“yes”} \mid \text{prompt (a) for statement } i). \tag{12}$$

where i denotes a statement and m denotes a model ($m \in \{1, \dots, n_m\}$, n_m being the number of models). Compare this with the human rating distribution in Equation 3 on page 2.

Similarly, the probability that the model thinks most people would agree with statement i is

$$d_i^{m,b} = p(\text{“yes”} \mid \text{prompt (b) for statement } i). \tag{13}$$

Figure S3 (top) on page 12 shows the frequencies of the human and model rating distributions. As depicted, human judgments are widely varied, with many statements receiving close to 50/50 ratings. For most models, on the other hand, these probabilities are predominantly driven to either 0 or 1. The same is observed for humans' and models' answers to question (b).

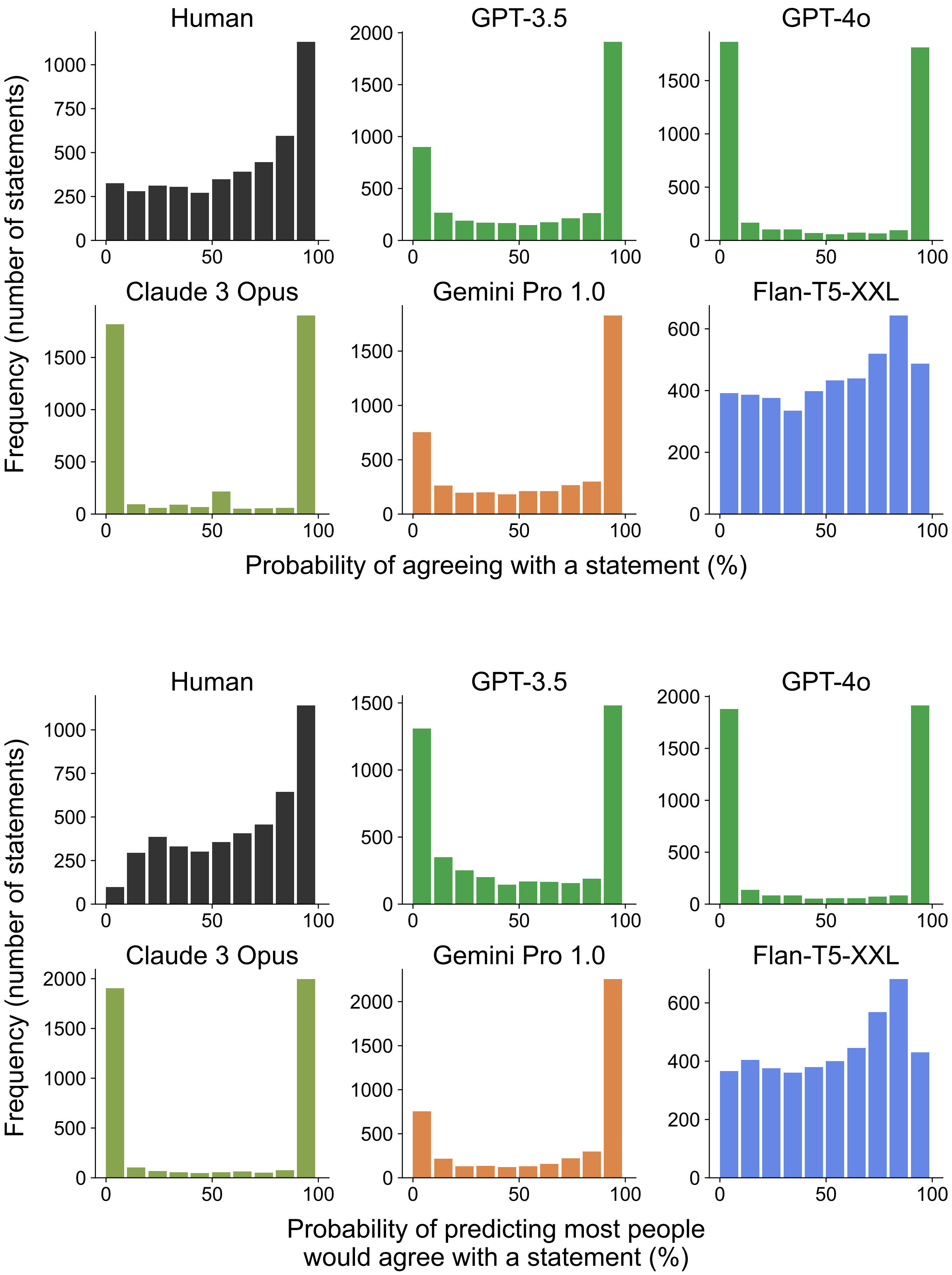

Figure S3. Top: frequencies of the human rating distribution, $d_i^{h,a}$ (Equation 3), and the model rating distribution $d_i^{m,a}$ (Equation 12) for some models. These are derived from responses to question (a). Bottom: distribution of the same quantities, but for question (b).

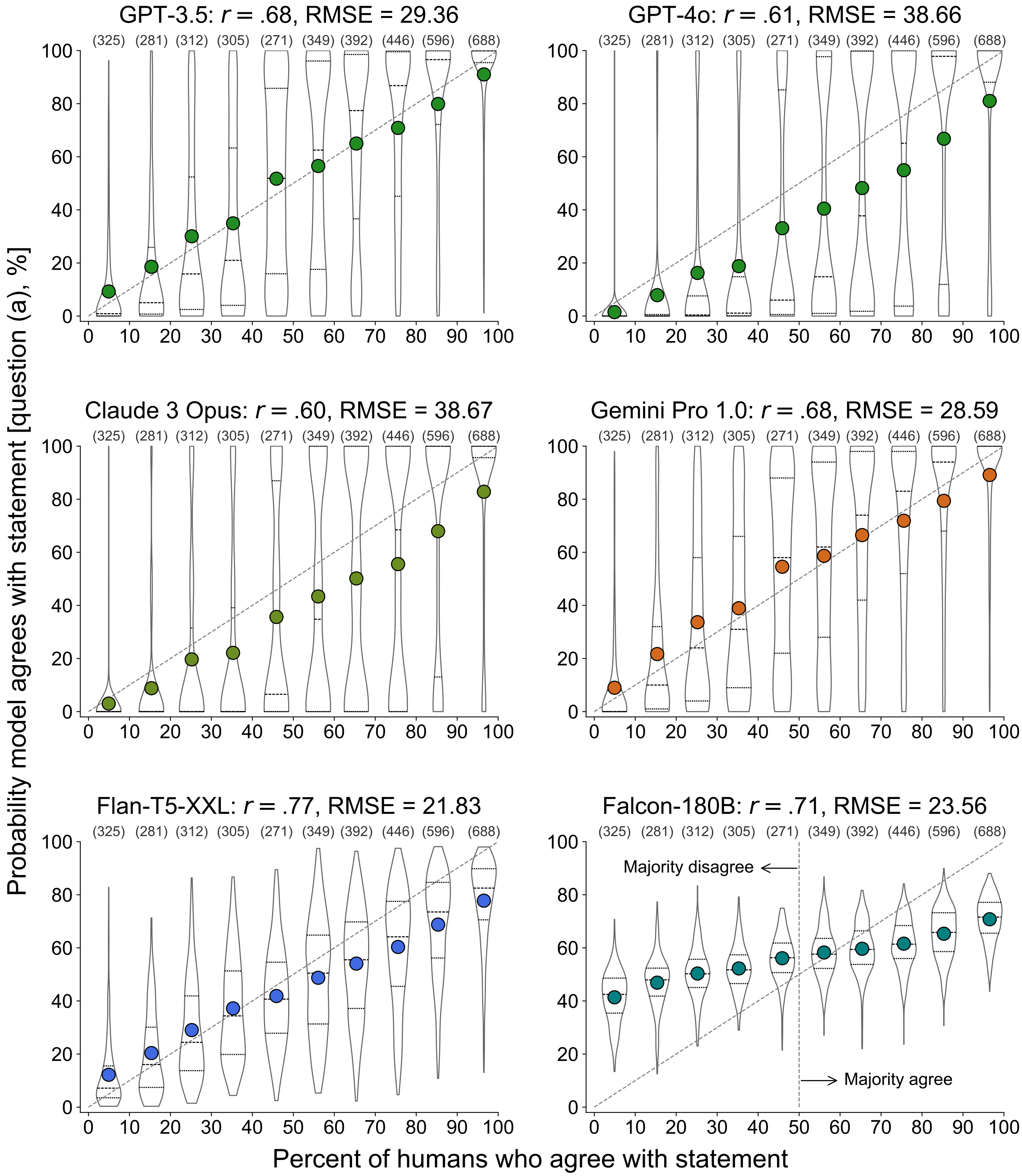

Figure S4. Relationship between human and model ratings of statements. The *x*-axis is the percentage of humans who expressed that they agree with a statement, divided into ten intervals. A value of at least 50% on the *x*-axis means that the majority of humans agree with the statement. The *y*-axis is the probability that an LLM agrees with a statement, in response to question (a): "Do you agree with this statement?" For each violin plot, the number on top and in parentheses is the number of statements in the corresponding interval; the three horizontal lines within the plot from top to bottom depict the 75th, 50th and 25th percentiles of the distribution; and the point within the plot shows the average y-value for statements within each bin. Also shown on top of each subplot are the Pearson correlation r (all significant with $p < .001$) and the root mean squared error (RMSE).

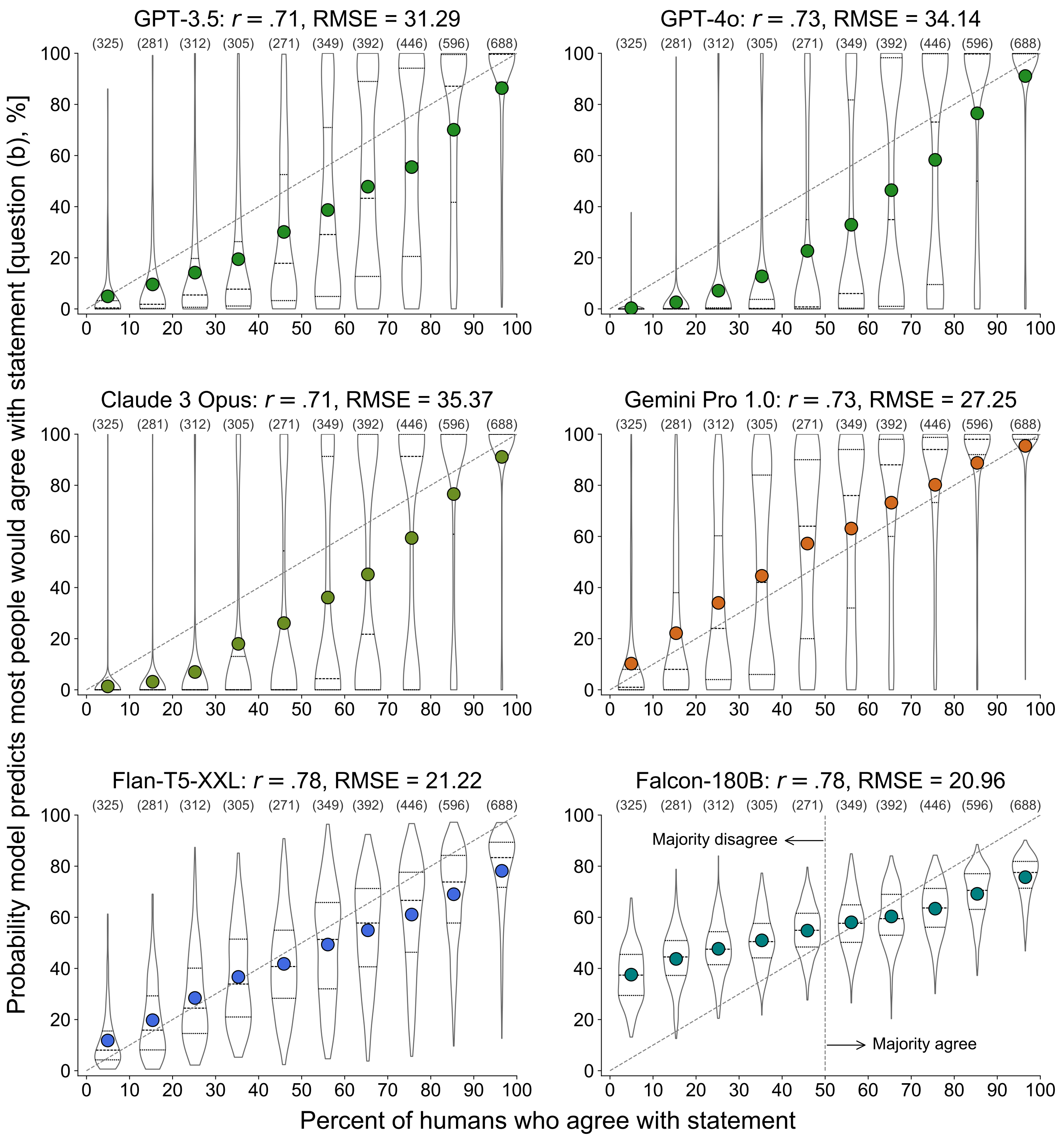

Figure S5. Relationship between human and model ratings of statements. The *x*-axis is the percentage of humans who expressed that they agree with a statement, divided into ten intervals. A value of at least 50% on the *x*-axis means that the majority of humans agree with the statement. The *y*-axis is an LLM's predicted probability that most people would agree with the statement, in response to question (b): "Do you think most people would agree with this statement?" For each violin plot, the number on top and in parentheses is the number of statements in the corresponding interval; the three horizontal lines within the plot from top to bottom depict the 75th, 50th and 25th percentiles of the distribution; and the point within the plot shows the average y-value for statements within each bin. Also shown on top of each subplot are the Pearson correlation r (all significant with $p < .001$) and the root mean squared error (RMSE).

Source	Statement	Number (%) of models
Aphorisms	Life is not separate from death it only seems that way.	19 / 35 (54.3%)
Aphorisms	One must discard the prejudice that the truth must be something tangible.	18 / 35 (51.4%)
News media	Public private partnerships should be adopted as a more important mechanism by congress.	18 / 35 (51.4%)
Aphorisms	We will bankrupt ourselves in the vain search for absolute security.	17 / 35 (48.6%)
Aphorisms	Philosophy is properly home sickness the wish to be everywhere at home.	17 / 35 (48.6%)
Aphorisms	The sage constantly has no set mind those who are good he regards as good and those who are not good he also regards as good in this way he attains goodness.	17 / 35 (48.6%)
Categorical response	The conflating of religion and god has been damning to humanity.	16 / 35 (45.7%)
Aphorisms	The miracles of earth are the laws of heaven.	15 / 35 (42.9%)
Aphorisms	Politeness has been defined to be artificial good nature but we may affirm with much greater propriety that good nature is natural politeness.	15 / 35 (42.9%)
Aphorisms	Live your life as though your every act were to become a universal law.	15 / 35 (42.9%)
Categorical response	When someone leaves a fast food store with a bag the bag will be full of food.	15 / 35 (42.9%)
Aphorisms	We act as though comfort and luxury were the chief requirements of life when all that we need to make us happy is something to be enthusiastic about.	15 / 35 (42.9%)
Campaign email	We should sign up for the mailing list.	15 / 35 (42.9%)
Aphorisms	Blessed is he who has found his work let him ask no other blessedness.	14 / 35 (40.0%)
Aphorisms	Chaos often breeds life when order breeds habit.	14 / 35 (40.0%)
Atomic	If max accidentally shot then max wants to call the ambulance.	14 / 35 (40.0%)
Aphorisms	Remember that life is neither pain nor pleasure it is serious business to be entered upon with courage and in a spirit of self sacrifice.	14 / 35 (40.0%)
News media	Governments should offer a path to citizenship for millions of illegal immigrants.	14 / 35 (40.0%)
Aphorisms	Doubt is a pain too lonely to know that faith is his twin brother.	14 / 35 (40.0%)
Campaign email	Lizzie fletcher will be a effective leader.	14 / 35 (40.0%)
Campaign email	We should support abortion and woman's right to choose.	14 / 35 (40.0%)
Campaign email	We should vote an underdog who supports a more civil government.	14 / 35 (40.0%)
Categorical response	No one religion is the right path.	14 / 35 (40.0%)
Aphorisms	Solitude produces originality bold and astonishing beauty poetry but solitude also produces perverseness the disproportionate the absurd and the forbidden.	14 / 35 (40.0%)
Aphorisms	The sage treasures everything so nothing is wasted.	13 / 35 (37.1%)

Table S4. Statements that receive diverging answers to questions (a) and (b) from LLM. For each statement, every LLM is asked two questions: (a) "Do you agree with this statement?" and (b) "Do you think most people would agree with this statement?" Most of the time, models give the same answer (either both "yes" or both "no") to these questions. In a minority of cases (about 10%), we observe the opposite, where one question receives "yes" and the other "no." This table shows the 25 statements that receive such divergence from the most models.

C Measuring Individual-Level Commonsensicality in LLMs

In this section, we consider each language model as a hypothetical human survey respondent, just like those described in Section A.2.

Start from $d_i^{m,a}$ (Equation 12), which is the probability that the model agrees with statement i, via its answer to question (a). We binarize this probabilistic output by taking its mode

$$\alpha_i^m = \mathbb{1}[d_i^{m,a} \geq 0.5]. \tag{14}$$

In other words, we consider that the model agrees with statement i (*i.e.*, $\alpha_i^{m,a} = 1$) if and only if it agrees with this statement with a probability of at least 0.5.

Similarly, the model believes that most people would agree with this statement if and only if it answers "yes" to question (b) with a probability of at least 0.5:

$$\beta_i^m = \mathbb{1}[d_i^{m,b} \geq 0.5]. \tag{15}$$

Based on these two binary answers, the model's *consensus* and *awareness* scores, respectively, are

$$C_m = \frac{1}{n_s} \sum_{i=1}^{n_s} \mathbb{1}\left[\alpha_i^m = \texttt{majority}_i^h\right], \tag{16}$$

$$A_m = \frac{1}{n_s} \sum_{i=1}^{n_s} \mathbb{1}\left[\beta_i^m = \texttt{majority}_i^h\right], \tag{17}$$

where $\texttt{majority}_i^h$ is the human majority rating for statement i (Equation 4). Compare these calculations with the consensus and awareness scores for humans, in Equations 9 and 10.

Finally, similar to Equation 11, the model's *commonsensicality* score is the geometric mean of its consensus and awareness scores:

$$M_m = \sqrt{C_m \times A_m}. \tag{18}$$

All three scores are between 0 and 1, and higher values indicate closer proximity to absolute common sense. The goal of this calculation is to compare M_m with M_j, the commonsensicality of person j defined in Equation 11.

Table S5 shows every model's consensus, awareness and commonsensicality scores.

C.1 Model Size and Commonsensicality

To investigate the relationship between a model's size and its commonsensicality score, we select from Table S3 (on page 9) 6 model families: Falcon, Flan-T5, Gemma, LLaMA-2/3, Mistral and Qwen2. Our selection criterion is that the family must include at least 2 models for which we know the sizes. See Table S5. We also exclude Gemini 1.0 Pro in the Gemma/Gemini family and Mistral-Large in the Mistral family because these models are proprietary with unknown sizes. For GPT, although we know that GPT-3.5 has 175 billion parameters, we do not know this information for GPT-4 and GPT-5.

These 6 families give us 23 models in total. Since model commonsensicality varies substantially across families, we perform a mixed-effects regression analysis to analyze the relationship between model size and commonsensicality. In R's syntax, the model is described by the following formula:

$$\texttt{commonsensicality} \sim \log_{10}(\texttt{model size}) + (1 \mid \texttt{model family}). \tag{19}$$

We also log-transform model size since these models can be orders of magnitude different from each other. In addition, we establish the 95% confidence interval of the effect of (log) model size using the `confint` function in R.

C.2 Model General Performance on LMArena and Commonsensicality

There are numerous general benchmarks of LLMs available publicly. We choose the LMArena benchmark (*23*) to represent a model's general performance, relative to other models.

LMArena compares 230 models side by side, which include 24 models in our collection. When a user visits its website,[†] two models are randomly chosen. The user then interacts with these models simultaneously, asking the same question and getting corresponding answers from both. Then the user decides whether they prefer the left or the right model. (They also can choose to tie them, or to indicate both models are bad.)

Models in this collection are then assigned an Elo score, with higher values indicating a higher chance of being preferred by humans. Essentially, the Elo scores are used to approximate the win-rate matrix, and these scores are estimated by Chiang *et al.* (*23*) using the Bradley-Terry maximum likelihood method.

The Elo score by LMArena can be interpreted as follows. Suppose two models, a and b, receive their respective Elo scores of R_a and R_b. Then the probability that model a is chosen over model b is

$$\frac{1}{1 + 10^{\frac{R_b - R_a}{400}}}. \tag{20}$$

As long as $R_a < R_b$, the denominator is greater than 2, the fraction is less than 0.5, and thus model a is preferred less frequently than model b. For example, GPT-3.5 (model a) and GPT-4-0125 (model b) are assigned Elo scores of $R_a = 1,224$ and $R_b = 1,314$, respectively. The probability that GPT-3.5 (a) is preferred over GPT-4-0125 (b) is

$$\frac{1}{1 + 10^{\frac{R_b - R_a}{400}}} = \frac{1}{1 + 10^{1,314 - 1,224}} \approx 0.37.$$

In other words, GPT-4o is expected to win about $100\% - 37\% = 63\%$ of the time. Elo scores are invariant to translation, *i.e.*, if all scores shift up or down by the same amount, the interpretation above does not change. In this paper, we keep the original scores that are reported on LMArena.

Since this calculation is updated over time—as more models are added to the benchmark and more users participate in evaluation—we report the scores that are the most updated at the time of writing, August 16, 2025. Models' Elo scores are calculated based on 3,866,847 pairwise comparisons.

Finally, for the 24 models in our collection that also exist on LMArena (see Table S5), we also extract their commonsensicality scores and evaluate the Pearson correlation between the two types of scores.

C.3 Model Performance on Static Commonsense Benchmark and Commonsensicality

In addition to the LMArena benchmark, we compare an LLM's commonsensicality with its performance on the OpenBookQA benchmark (*24*). OpenBookQA is a set of multiple-choice questions generated based on 1,326 facts: Amazon Mechanical Turk workers were given a fact, such as "poison causes harm to living things," and then asked to author a question using that fact. There are 5,957 questions in this dataset, which are split into three datasets: training (4,957 questions), development (500), and test (500). Below are some examples of these questions.

```
Example question:  Poison causes harm to which of the following?
A. A tree.
B. A robot.
C. A house.
D. A car.
(Fact:  Poison causes harm to living things.)

Example question:  A magnet would attract a
A. wooden hairbrush.
```

[†]LMArena can be accessed at https://lmarena.ai. Previously, it was called LMSYS Arena and could be accessed at https://chat.lmsys.org.

Model	Consensus	Awareness	Commonsensicality	Size	LMArena	OpenBookQA
Claude 3 Haiku	58.8	64.1	61.4	–	1,262	85.4
Claude 3 Sonnet	60.9	62.2	61.5	–	1,282	91.8
Claude 3 Opus	73.4	77.4	75.4	–	1,322	95.6
DBRX	73.7	79.0	76.3	132	1,198	91.0
Falcon-7B	66.6	66.1	66.3	7	–	26.0
Falcon-40B	73.0	77.2	75.1	40	–	66.2
Falcon-180B	78.6	81.3	79.9	180	1,148	–
Flan-T5-Small	34.4	33.9	34.2	.08	–	–
Flan-T5-Base	56.8	59.5	58.1	.25	–	–
Flan-T5-Large	77.3	76.5	76.9	.78	–	–
Flan-T5-XL	73.3	72.7	73.0	3	–	–
Flan-T5-XXL	79.9	80.9	80.4	11	–	–
Gemma-2B	65.2	66.6	65.9	2	1,093	–
Gemma-7B	73.2	70.9	72.0	7	1,136	80.8
Gemini Pro 1.0	78.4	81.1	79.7	–	1,221	78.8
GPT-3.5	78.3	75.4	76.8	175	1,224	83.8
GPT-4-0125	77.6	79.2	78.4	–	1,314	–
GPT-4-0409	78.0	83.3	80.6	–	1,324	97.0
GPT-4o	72.5	77.9	75.2	–	1,441	96.6
GPT-5	71.9	79.6	75.7	–	1,461	–
LLaMA-2-7B	74.0	76.0	75.0	7	1,113	54.4
LLaMA-2-13B	48.5	44.5	46.5	13	1,147	63.4
LLaMA-2-70B	65.7	61.4	63.5	70	1,175	83.8
LLaMA-3-8B	57.2	66.5	61.7	8	1,225	76.6
LLaMA-3-70B	72.0	76.8	74.4	70	1,276	93.4
Mistral-7B	80.2	80.7	80.4	7	1,155	77.6
Mixtral-8x7B	77.8	75.0	76.4	47	1,201	86.8
Mixtral-8x22B	80.7	84.0	82.3	141	1,232	88.2
Mistral-Large	80.4	82.2	81.3	–	1,244	89.4
OLMo-7B	74.3	71.0	72.7	7	1,080	22.2
Qwen2-0.5B	67.1	66.5	66.8	.5	–	–
Qwen2-1.5B	75.4	73.8	74.6	1.5	–	–
Qwen2-7B	79.7	81.1	80.4	7	–	–
Qwen2-57B	80.4	81.4	80.9	57	–	–
Qwen2-72B	80.5	81.8	81.1	72	1,264	95.4

Table S5. Individual-level consensus (C_m, Equation 16), awareness (A_m, Equation 17) and commonsensicality (M_m, Equation 18) scores for all models. This table also presents the sizes of these models (in billions of parameters, also shown in Table S3) as well as their Elo scores (updated on August 16, 2025) on the LMArena benchmark (*23*) and their accuracy on the OpenBookQA benchmark (*24*).

```
B. Oranges.
C. Glassware.
D. Zippers.
(Fact: A magnet attracts ferromagnetic metals through magnetism.)

Example question: As a car approaches you in the night,
A. the headlights remain at a constant.
B. the headlights turn off.
C. the headlights become more intense.
D. the headlights recede into the dark.
(Fact: As a source of light becomes closer, that source will appear brighter.)
```

Here we only use the test set with 500 questions. The human performance on this subset is reported to be 91.7%. For a standardized evaluation setting, we refer to the Holistic Evaluation of Language Models (HELM) framework (*25*, version 1.13.0, released January 10, 2025), and find that 22 out of 35 models that we test in this paper are present on this framework. For each question in the test set, HELM prompts every LLM in a 5-shot fashion, *i.e.*, the model is given 5 questions with their corresponding correct answers first, and then the focal test question at the end without the answer. The job of the model is to output a letter, `A`, `B`, `C`, or `D`, for the focal question. Below is an example.

```
The following are multiple choice questions (with answers) about common sense.

Question: Your body goes into starvation mode when you have insufficient amounts
of
A. shoes
B. fun
C. pants
D. sustenance
Answer: D

Question: What cells can perform photosynthesis?
A. animals
B. inorganic minerals
C. flora
D. critters
Answer: C

Question: What does the digestive system break down into simple substances?
A. metals
B. stones
C. plastic food
D. nutriment
Answer: D

Question: evaporation is the first stage in the what cycle
A. H2O
B. lunar
C. growth
D. menstrual
```

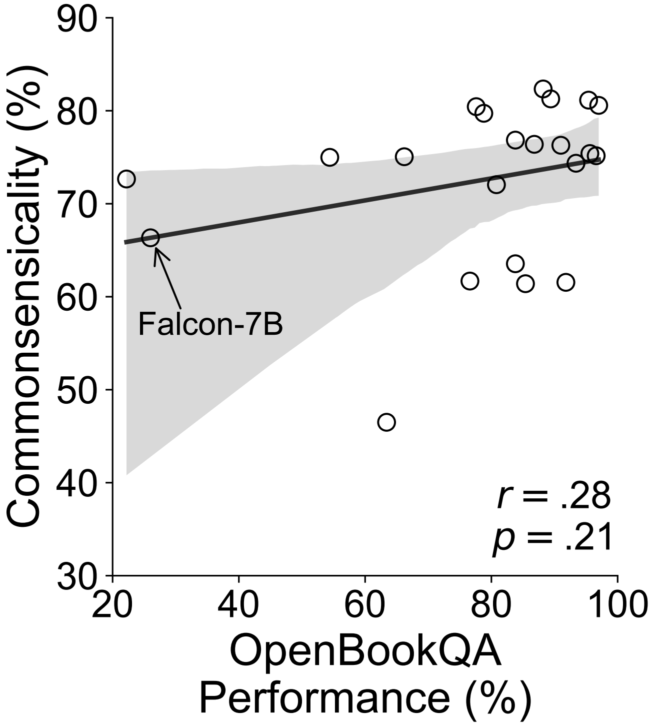

Figure S6. Correlation between an LLM's performance on the OpenBookQA benchmark and its commonsensicality. A total of 22 models are found on the HELM framework. Pearson correlation and the two-sided *p*-value are reported. Also shown is the best-fit line and its 95% confidence interval.

```
Answer: A

Question: A fire started in a forest but it wasn't started by people. What could
have been the cause?
A. a careless bird
B. a smoking bear
C. electricity
D. a campfire
Answer: C

Question: A person wants to start saving money so that they can afford a nice
vacation at the end of the year. After looking over their budget and expenses,
they decide the best way to save money is to
A. make more phone calls
B. quit eating lunch out
C. buy less with monopoly money
D. have lunch with friends
Answer:
```

HELM then determines that the model is correct if and only if it outputs the letter `B` in its answer. A model's accuracy is the percentage of times it is correct across all 500 test questions. This performance is reported in Table S5.

Finally, Figure S6 plots the relationship between a model's accuracy on OpenBookQA and its commonsensicality for the 22 models found on HELM. The Pearson correlation and its p-value are reported, as well as the best-fit line and its 95% confidence interval.

	Consensus		Awareness		Commonsensicality	
	(I)	(II)	(I)	(II)	(I)	(II)
Claude 3 Haiku	58.8 (31)	60.5 (31)	64.1 (30)	65.5 (30)	61.4 (32)	63.0 (32)
Claude 3 Sonnet	60.9 (30)	62.6 (30)	62.2 (31)	63.7 (31)	61.5 (31)	63.1 (31)
Claude 3 Opus	73.4 (19)	75.1 (20)	77.4 (15)	78.2 (15)	75.4 (17)	76.6 (17)
DBRX	73.7 (18)	75.2 (18)	79.0 (13)	79.9 (13)	76.3 (15)	77.5 (14)
Falcon-7B	66.6 (27)	68.1 (27)	66.1 (29)	67.7 (28)	66.3 (27)	67.9 (27)
Falcon-40B	73.0 (22)	74.8 (22)	77.2 (16)	77.9 (17)	75.1 (19)	76.3 (20)
Falcon-180B	78.6 (8)	80.3 (8)	81.3 (6)	82.5 (5)	79.9 (9)	81.4 (9)
Flan-T5-Small	34.4 (35)	36.1 (35)	33.9 (35)	35.6 (35)	34.2 (35)	35.8 (35)
Flan-T5-Base	56.8 (33)	58.6 (33)	59.5 (33)	60.6 (33)	58.1 (33)	59.6 (33)
Flan-T5-Large	77.3 (14)	78.9 (14)	76.5 (18)	77.9 (16)	76.9 (12)	78.4 (12)
Flan-T5-XL	73.3 (20)	75.0 (21)	72.7 (23)	74.1 (23)	73.0 (23)	74.6 (23)
Flan-T5-XXL	79.9 (6)	81.6 (6)	80.9 (9)	82.1 (8)	80.4 (7)	81.8 (6)
Gemma-2B	65.2 (29)	66.8 (29)	66.6 (26)	67.9 (27)	65.9 (28)	67.3 (28)
Gemma-7B	73.2 (21)	75.1 (19)	70.9 (25)	72.3 (25)	72.0 (25)	73.7 (25)
Gemini Pro 1.0	78.4 (9)	80.0 (9)	81.1 (7)	82.2 (7)	79.7 (10)	81.1 (10)
GPT-3.5	78.3 (10)	80.0 (10)	75.4 (20)	76.3 (20)	76.8 (13)	78.1 (13)
GPT-4-0125	77.6 (13)	79.1 (13)	79.2 (12)	80.2 (12)	78.4 (11)	79.7 (11)
GPT-4-0409	78.0 (11)	79.4 (11)	83.3 (2)	84.2 (2)	80.6 (5)	81.8 (7)
GPT-4o	72.5 (23)	74.2 (23)	77.9 (14)	78.8 (14)	75.2 (18)	76.4 (18)
GPT-5	71.9 (25)	73.5 (25)	79.6 (11)	80.6 (11)	75.7 (16)	77.0 (16)
LLaMA-2-7B	74.0 (17)	75.6 (17)	76.0 (19)	77.2 (19)	75.0 (20)	76.4 (19)
LLaMA-3-8B	57.2 (32)	58.9 (32)	66.5 (27)	67.7 (28)	61.7 (30)	63.2 (30)
LLaMA-2-13B	48.5 (34)	50.3 (34)	44.5 (34)	46.1 (34)	46.5 (34)	48.1 (34)
LLaMA-2-70B	65.7 (28)	67.4 (28)	61.4 (32)	62.4 (32)	63.5 (29)	64.9 (29)
LLaMA-3-70B	72.0 (24)	73.6 (24)	76.8 (17)	77.8 (18)	74.4 (22)	75.7 (22)
Mistral-7B	80.2 (5)	81.7 (5)	80.7 (10)	82.1 (9)	80.4 (6)	81.9 (5)
Mixtral-8x7B	77.8 (12)	79.4 (12)	75.0 (21)	75.7 (21)	76.4 (14)	77.5 (15)
Mixtral-8x22B	80.7 (1)	82.2 (1)	84.0 (1)	84.9 (1)	82.3 (1)	83.6 (1)
Mistral-Large	80.4 (4)	82.0 (4)	82.2 (3)	83.4 (3)	81.3 (2)	82.7 (2)
OLMo-7B	74.3 (16)	75.9 (16)	71.0 (24)	72.3 (24)	72.7 (24)	74.1 (24)
Qwen2-0.5B	67.1 (26)	68.7 (26)	66.5 (27)	67.9 (26)	66.8 (26)	68.3 (26)
Qwen2-1.5B	75.4 (15)	76.8 (15)	73.8 (22)	74.9 (22)	74.6 (21)	75.8 (21)
Qwen2-7B	79.7 (7)	81.2 (7)	81.1 (8)	82.0 (10)	80.4 (8)	81.6 (8)
Qwen2-57B	80.4 (3)	82.1 (2)	81.4 (5)	82.4 (6)	80.9 (4)	82.3 (4)
Qwen2-72B	80.5 (2)	82.1 (3)	81.8 (4)	82.8 (4)	81.1 (3)	82.5 (3)
Spearman's r	.9989		.9974		.9986	

Table S6. Individual-level consensus, awareness and commosensicality for LLMs under two calculation variants. In variant (I), the majority rating for each statement i follows Equation 4, based only on the *human* ratings. In variant (II), the majority rating follows Equation 21, where the determination of the majority also takes into account the model's rating. The numbers in parentheses indicate the ranking of these scores within their respective column. Spearman's ranking correlation r is calculated by comparing variants (I) and (II) for each type of score. All correlations are significant with p ¡ .00001.

C.4 Comparing Models and Humans Side by Side on Commonsensicality

When comparing a model and a human directly on their commonsensicality scores, note that each person was only tasked with labeling 50 randomly chosen statements, whereas a model labels all 4,407 of them. For a fair comparison, we restrict the calculation of consensus, awareness and commonsensicality of the model only to the 50 statements that the person labeled.

Specifically, for person j the calculations in Equations 9 to 11 (6) do not change because they are already with respect to Φ_j, the subset of statements that were labeled by j. To compare model m with person j, we modify Equations 16 and 17 above so that the average is with respect to Φ_j:

$$C_m = \frac{1}{|\Phi_j|} \sum_{i \in \Phi_j} \mathbb{1}\left[\alpha_i^m = \texttt{majority}_i^h\right],$$

$$A_m = \frac{1}{|\Phi_j|} \sum_{i \in \Phi_j} \mathbb{1}\left[\beta_i^m = \texttt{majority}_i^h\right].$$

C.5 Alternative Calculation of Model Commonsensicality with Model-Contributed Majority Vote

In calculating the model commonsensicality, we rely on the majority rating of each statement; see Equation 4 (page 5). When each LLM is treated as a survey participant, its rating can also contribute to this majority vote. Here, we explore this alternative calculation to see whether model commonsensicality can be sensitive to it.

Formally, for each model m, define the human-plus-model majority rating for statement i as

$$\texttt{majority}_i^h = \mathbb{1}\left[\frac{1}{|\Omega_i| + 1}\left(\alpha_i^m + \sum_{j \in \Omega_i} A_{i,j}\right) \geq 0.5\right], \tag{21}$$

where Ω_i is the set of humans that were assigned to rate statement i; α_i^m is the model's binary rating for the statement in response to question (a) from Equation 14 (page 16); and A is the matrix of human ratings for all statements from Equation 1 (page 2).

Table S6 on page 21 compares the consensus, awareness and commonsensicality scores for each model with respect to the two calculation variants. While their raw scores change, the models' relative rankings do not significantly change, as evidenced by the Spearman correlation coefficients at the bottom of the table.

D Measuring Commonsensicality when LLMs are Simulators of Silicon Samples

In this section, we use each language model not as an individual survey participant (*cf.* Section C), but rather as a generator of "silicon samples" which, together, form a hypothetical population of raters. Common sense in this case is measured with respect to this entire population, rather than for each silicon sample within.

To make this clearer, imagine a population of silicon samples, each represented by the same language model. When we ask each respondent in this population questions (a) and (b), we record their binary answers ("yes" or "no"). Each respondent is treated as independent from the others, and so collecting answers for this entire population amounts to resampling the answers to the same questions. In the limit—*i.e.*, if the size of the population goes to infinity—the proportion of respondents that agree with statement i is exactly the probability with which the language model agrees with this statement, $d_i^{m,a}$ (defined in Equation 12 on page 11). Similarly, the proportion of silicon respondents that believe most people would agree with this statement is exactly $d_i^{m,b}$ (Equation 13 on page 11).

Mimicking the calculations in Section A.1, we first define the majority rating within this population of silicon samples for statement i as

$$\texttt{majority}_i^m = \mathbb{1}[d_i^{m,a} \geq 0.5]. \tag{22}$$

The consensus score of statement i with respect to this population is

$$c_i^m = 2 \times |d_i^{m,a} - 0.5|. \tag{23}$$

The statement's awareness score is the proportion of silicon samples that accurately predict the majority opinion via their answer to question (b):

$$a_i^m = \begin{cases} d_i^{m,b} & \text{if } \texttt{majority}_i^m = 1 \\ 1 - d_i^{m,b} & \text{if } \texttt{majority}_i^m = 0. \end{cases} \tag{24}$$

Which leads to its commonsensicality score of

$$m_i^m = \sqrt{c_i^m \times a_i^m}.. \tag{25}$$

The objective is to compare m_i^m with m_i^h (Equation 8 on page 6). First, we look at the correlation between these two scores, taken over $n_s = 4,407$ statements. Specifically, we calculate the Pearson correlation between m_i^m with m_i^h as well as its two-sided p-values (after Bonferroni correction).

We also make this comparison through a different lens: how well does a statement's commonsensicality score in the population of silicon samples predict its same score in the population of humans? To do so, we fit a linear regression model, predicting m_i^h using m_i^m. We report the regression coefficient as well as its two-sided p-values.

D.1 Comparing Statement Commonsensicality Scores within Humans and Silicon Samples by Statement Type

Each statement in our corpus was labeled by MTurk workers according to six epistemological features:

1. *Behavior*
    - *Social* (*N* = 2,939): it refers to beliefs, perceptions, preferences, and socially constructed rules that govern human experience; it can be "real" or opinion, but is intrinsically of human origins. *e.g.*, "I exist and am the same person I was yesterday," "He yelled at me because he was angry," "There are seven days in the week."
    - *Physical* (*N* = 1,468): it refers to objective features of the world as described by, say, physics, biology, engineering, mathematics or other natural rules; it can be measured empirically, or derived logically. *e.g.*, "Men on average are taller than women," "The Earth is the third planet from the Sun," "Ants are smaller than Elephants."

2. *Everyday*
    - *Everyday* (*N* = 2,779): people encounter, or could encounter, situations like this in the course of their ordinary, everyday experiences, *e.g.*, "Touching a hot stove will burn you," "Commuting at rush hour takes longer," "It is rude to jump the line."
    - *Abstract* (*N* = 1,628): this claim refers to regularities or conclusions that cannot be observed or arrived at solely through individual experience, *e.g.*, "Capitalism is a better economic system than Communism," "Strict gun laws save lives," "God exists."
3. *Figure of speech*
    - *Figure of speech* (*N* = 854): it contains an aphorism, metaphor, hyperbole, *e.g.*, "Birds of a feather flock together," "A friend to all is a friend to none."
    - *Literal language* (*N* = 3,553): it is plain and ordinary language that means exactly what it says. e.g. "The sky is blue," "Elephants are larger than dogs," "Abraham Lincoln was a great president."
4. *Judgment*
    - *Normative* (*N* = 3,191): it refers to a judgment, belief, value, social norm or convention. *e.g.*, "If you are going to the office, you should wear business attire, not a bathing suit," "Treat others how you want them to treat you," "Freedom is a fundamental human right."
    - *Positive* (*N* = 1,216): it refers to something in the world such as an empirical regularity or scientific law, *e.g.*, "Hot things will burn you," "The sun rises in the east and sets in the west."s
5. *Opinion*
    - *Opinion* (*N* = 2,805): it is something that someone might think is true, or wants others to think is true, but can't be demonstrated to be objectively correct or incorrect; it is inherently subjective. *e.g.*, "FDR was the greatest US president of the 20th Century," "The Brooklyn Bridge is prettier than the Golden Gate," "Vaccine mandates are a tolerable imposition on individual freedom."
    - *Fact* (*N* = 1,602): it is something that can be demonstrated to be correct or incorrect, independently of anyone's opinion, *e.g.*, "the earth is the third planet from the sun," (this is correct and we know it is correct), "Obama was the 24th president of the United States" (this is incorrect, but we know it's incorrect) "It will be sunny next Tuesday" (we don't yet know if this is correct, but we will be able to check in the future).
6. *Reasoning*
    - *Knowledge* (*N* = 1,672): the claim refers to some observation about the world; it may be true or false, opinion or fact, subjective or objective *e.g.*, "The sun rises in the east and sets in the west," "Dogs are nicer than cats," "Glasses break when they are dropped."
    - *Reasoning* (*N* = 2,735): the claim presents a conclusion that is arrived at by combining knowledge and logic, *e.g.*, "The sun is in the east, therefore it is morning," "My dog is wagging its tail, therefore it is happy," "The glass fell off the table, therefore it will break and the floor will become wet."

For every dichotomy, the corpus of $n_s = 4,407$ statements is divided into two subsets corresponding to the two opposing dimensions in that dichotomy. Figure S8 compares the distributions of statement commonsensicality, computed using human ratings, with respect to each subset in every dichotomy.

Our goal is to compare the commonsensicality scores (either by humans or by models) between these two dimensions. For instance, a statement is either about a fact or an opinion, corresponding to dichotomy 5 above. Are statements depicting facts more commonsensical than those depicting opinions?

To do so, we compute the difference in the mean commonsensicality score between the two subsets of statements. This is done for every population: of humans and of silicon samples created by 35 models. To establish a confidence interval, we construct 1,000 bootstrap samples and calculate this difference for each sample.

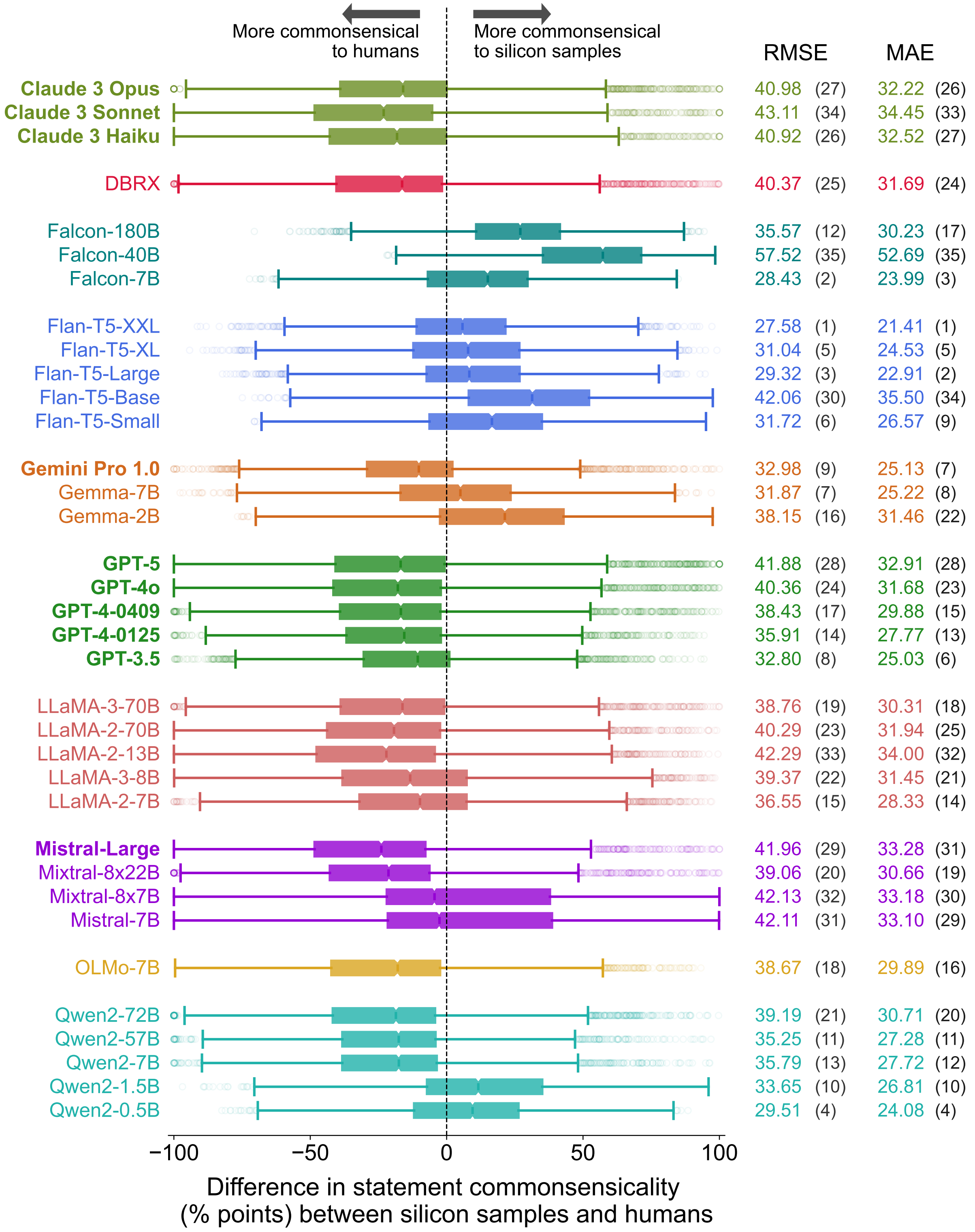

Figure S7. Comparison of statement commonsensicality scores between humans and silicon samples generated by LLMs. The *x*-axis represents the difference in a statement's scores between these two populations, where a positive difference indicates that the statement is more commonsensical to silicon samples than it is to humans (*i.e.*, $m_i^m > m_i^h$). Each box indicates the inter-quartile range, and the median notch is contained within it. The root mean-squared error (RMSE) and mean absolute error (MAE) are calculated based on the difference between m_i^m and m_i^h, averaged over 4,407 statements. The numbers in parentheses are the rankings of the RMSE and MAE (in ascending order); for example, Claude 3 Opus has an RMSE ranking of 27 out of 35 models, whereas its MAE ranking is 26 out of 35.

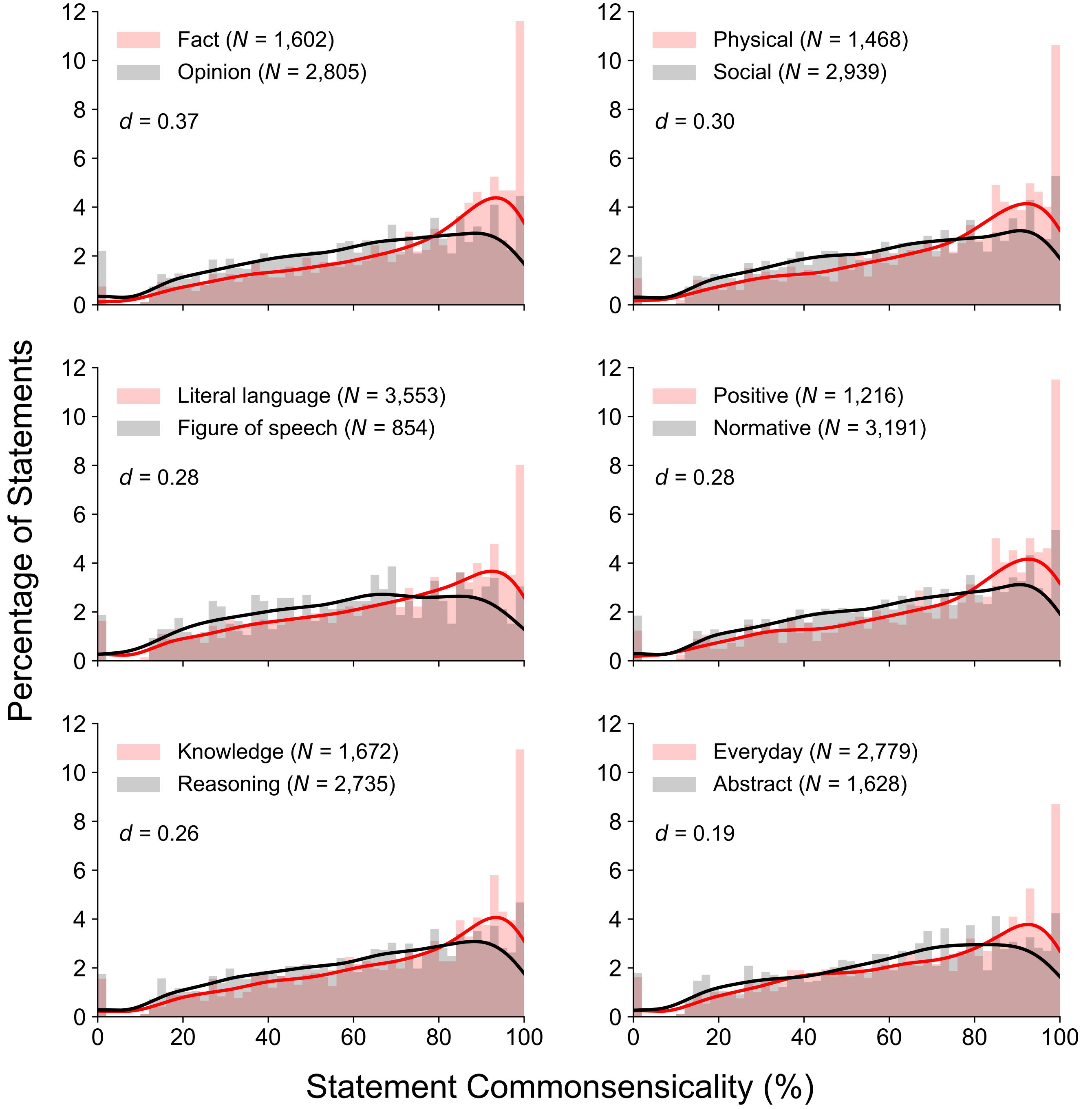

Figure S8. Comparison of statement commonsensicality with respect to each epistemological dichotomy. In each subplot, the corpus of 4,407 statements is split into two complementary subsets, such as facts and opinions. Within each subset, the distribution of statement commonsensicality is shown with a histogram and a kernel density estimation (KDE) line of the corresponding color. Also noted in each subplot is the standardized difference in means between the two (red and black) distributions.

E Other Variants of Model Evaluation Settings

In this section we present additional results of model ratings in response to several variants of the default prompt we use in the main paper.

E.1 Model's Reasoning Following Binary Answer

In Tables S7 and S8, we keep our original prompts and let GPT-4o continue sampling to get its full answer. The text following the first tokens—"yes" or "no"—typically represents the model's elaboration of its answer.

E.2 Model's Reasoning Before Binary Answer

In Tables S9 and S10, we slightly modify our prompts as follows. Instead of asking GPT-4o to start its answer with a "yes" or "no," we instruct it to explain what it thinks in a few sentences first.

E.3 Clarifying the Role of LLMs in the Survey

In designing our default prompts, we intend to blind the LLMs to the purpose of data collection. Specifically, we do not disclose who we are and why we would like to solicit their ratings of the given statements.

It is possible that in responding to our prompts, the models may not do so as an independent survey participant, but rather as a useful assistant that is trying to give an answer that satisfies its user. Here, we design a new prompt setting, which we call "role clarification," that emphasizes this aspect. Specifically, we use the following instruction in the system message: "You are an independent participant in a survey administered by academic researchers, who study commonsense beliefs. You will be presented with a statement, and asked a question about that statement. Answer the question independently, and please do not take into account what you think the researchers might want you to say."

We collect ratings for three models: GPT-3.5, GPT-4-0409 and GPT-4o. First, Figure S9 on page 32 presents the calibration of the models' output probabilities for both questions (a) and (b). Second, Table S11 on page 32 compares the evaluation results of these models under two settings: default and role clarification. In particular, we replicate our calculation of two metrics: individual common sense (measured by the commonsensicality score) and group common sense (measured by the Pearson correlation between the statement scores calculated from silicon samples generated by a model and the same statement scores arising from human ratings). Finally, Table S12 on page 33 shows some examples of GPT-4o's elaboration after its binary ratings.

(a) Do you agree with this statement? Start your answer with a "yes" or "no".	
**Statement: "Starting a new job people do what is expected of them for that job."**	
Yes. Starting a new job typically involves meeting the expectations set by the employer. New employees often try to understand and fulfill their roles and responsibilities to make a positive impression and secure their position. They might be motivated to demonstrate their capabilities, align with the company's goals, and adhere to the prescribed norms and procedures. However, this doesn't mean that every individual will perfectly meet these expectations from the start. There can be a learning curve, and the extent to which a new employee successfully meets job expectations can vary	No. While many people strive to meet or exceed expectations when starting a new job, this is not universally true for everyone. Various factors can influence an individual's performance, such as their understanding of the job requirements, the level of training and support they receive, their personal motivation and work ethic, and the overall work environment. Some individuals may exceed expectations due to high motivation and enthusiasm, while others might struggle to meet basic requirements due to inadequate onboarding, lack of clarity in their role, or personal challenges.
**Statement: "One must discard the prejudice that the truth must be something tangible."**	
Yes. The statement encourages us to broaden our understanding of truth beyond the confines of physical tangibility. While tangible evidence and sensory experiences are crucial in many contexts, especially in science and daily life, they do not encompass the entirety of what can be considered true. For example, mathematical truths, logical principles, and abstract concepts such as justice or love are not tangible, yet they hold significant validity and impact. Additionally, philosophical and metaphysical inquiries often deal with truths that are not accessible through sensory	No. The statement suggests that truth is not necessarily tied to tangible or physical entities. I tend to agree with this view. Truth can encompass abstract concepts such as love, justice, and mathematical principles, which are not physical but can be understood intellectually and emotionally. Moreover, many scientific truths describe principles that are not directly observable but are inferred through indirect evidence and logical reasoning, like the existence of subatomic particles or the concept of gravitational forces. Discarding the notion that truth must be tangible allows for
**Statement: "People open a window when they feel hot."**	
Yes, I agree with the statement to an extent. Many people do open a window when they feel hot as a way to allow fresh air to circulate and help cool down the indoor environment. However, this behavior can depend on various factors, such as the availability of air conditioning, the outside temperature, air quality, and individual preferences. For example, if it is very hot or humid outside, or if the air quality is poor, people might opt to use fans or air conditioning instead of opening a	No, I do not entirely agree with this statement. While it is true that some people may choose to open a window when they feel hot as a way to cool down, others might opt for different methods. These alternate methods can include using air conditioning, electric fans, or cool towels. The choice depends largely on individual preferences, availability of resources, and specific circumstances, such as outdoor temperatures and air quality. Therefore, while opening a window can be a common response to feeling hot, it is not
**Statement: "There is nothing more tragic in life than the utter impossibility of changing what you have done."**	
Yes. The statement highlights a poignant aspect of human existence: the permanence of past actions. This notion can indeed evoke a deep sense of tragedy, as it implies an irreversible impact of decisions that, in hindsight, one might wish to alter. The inability to change past mistakes can lead to feelings of regret and remorse, which are powerful emotions that can shape a person's future and mentality. However, it's important to consider the complexity of human experience. While the inability to change the past is unchangeable,	No. While the inability to change past actions can indeed be a source of deep regret and sorrow, it is not necessarily the most tragic aspect of life. Human resilience often allows individuals to find meaning and growth even in the face of irreversible mistakes. Additionally, the ability to learn from past actions and make amends, when possible, can transform personal tragedy into a catalyst for positive change and development. Life's tragedies come in many forms, and the impact of each varies based on personal context and perspective.

Table S7. Continuation in GPT-4o's ratings of some statements, in response to question (a) "Do you agree with this statement?" Some answers are not finished because we set a maximum number of generated tokens to 100.

(b) Do you think most people would agree with this statement? Start your answer with a "yes" or "no".	
**Statement: "The proliferation of charter schools affects the ability of states to efficiently distribute tax dollars."**	
Yes. Most people are likely to agree with this statement because the proliferation of charter schools can lead to a redistribution of public funding from traditional public schools to charter schools. This shift can complicate the way states allocate education budgets, potentially creating challenges in ensuring that all schools have sufficient resources to operate effectively. The presence of more charter schools may result in a need for adjustments in funding formulas and could lead to debates over equity and resource distribution among different types of schools. These factors make it reasonable to suggest that	No. Most people might not necessarily agree with the statement, as opinions on charter schools can vary widely depending on individual perspectives, political beliefs, and personal experiences. While some individuals may agree that charter schools impact the distribution of tax dollars by diverting funds from traditional public schools, others might argue that charter schools can offer innovative educational models and serve students more effectively, justifying their funding. The debate is multifaceted, and agreement on the statement is not universal.
**Statement: "One must discard the prejudice that the truth must be something tangible."**	
Yes. While opinions on this statement may vary, many people might agree that truth is not necessarily something tangible. Various philosophical, scientific, and experiential perspectives suggest that truth can encompass abstract concepts like mathematical truths, moral principles, or personal experiences that are not physically tangible but are nonetheless real and significant. This broader understanding of truth recognizes its multifaceted nature, transcending the limitations of physical tangibility.	No. While some people might agree with the statement, especially those with a philosophical or abstract outlook on truth, many others might find it challenging to accept that truth is not necessarily tangible. In general, people often rely on tangible evidence and concrete experiences to verify and understand truths. This is deeply ingrained in scientific thinking and everyday reasoning, where physical proof and observable data play crucial roles. Therefore, while the sentiment encourages open-mindedness and the consideration of abstract or conceptual truths, it may not resonate with
**Statement: "Test your ideas follow the evidence and always remember that you could be wrong."**	
Yes. Most people would likely agree with this statement because it underscores the importance of critical thinking, openness to new information, and intellectual humility. Testing ideas and following evidence are fundamental principles of scientific inquiry and rational discourse. Recognizing that one could be wrong fosters a mindset conducive to learning and growth, encouraging individuals to remain flexible and adaptable in their beliefs and judgments. While not everyone may consistently practice these principles, the general sentiment is widely supported in educational, professional, and personal development contexts.	No. While the statement espouses a scientific and open-minded approach to understanding the world, many people may struggle with or resist the implications. Personal biases, emotional investments, cognitive dissonance, and societal norms can often hinder individuals from fully embracing the idea that they could be wrong. People may also find it challenging to rigorously test their ideas and follow evidence, especially when it contradicts deeply held beliefs or values. Therefore, while the philosophy behind the statement is fundamentally sound, widespread agreement and application
**Statement: "If you want to take finals then you should attend a class."**	
Yes. Most people would likely agree with the statement, "If you want to take finals then you should attend a class," because attending classes is generally considered essential for understanding the course material and being well-prepared for finals. Regularly attending classes allows students to keep up with the curriculum, participate in discussions, ask questions, and receive guidance from instructors—all of which contribute to better performance in exams. Therefore, this statement aligns with common educational expectations and practices.	No. don't think most people would agree with the statement as it is. The connection between attending class and taking finals is not explicitly clear or universally applicable. Different educational systems and institutions may have varying requirements. While attending classes generally helps in better understanding the material and performing well in finals, it isn't always a strict precondition for being allowed to take the exams. For a statement to be more broadly agreeable, it might need to be rephrased to emphasize the benefits of attending classes in relation to

Table S8. Continuation in GPT-4o's ratings of some statements, in response to question (b) "Do you think most people would agree with this statement?" Some answers are not finished because we set a maximum number of generated tokens to 100.

**(a) Do you agree with this statement? Please explain what you think in a few sentences, then end our answer with "So the answer is yes." or "So the answer is no."**	
**Statement: "Starting a new job people do what is expected of them for that job."**	
I believe that the statement is generally true. Most individuals start a new job with the intention of doing what is expected of them because they want to make a good first impression, fulfill their role requirements, and secure their position within the company. They tend to follow guidelines and meet expectations as they are eager to prove their competency and adapt to the new work environment. So the answer is yes.	Whether people do what is expected of them when starting a new job can depend on several factors, including the clarity of job expectations, the individual's level of experience, and the organizational culture. While many people strive to meet or exceed job expectations to make a good impression, there can also be a learning curve and potential misunderstandings about what is required. Thus, it is not a universal certainty that all new hires will automatically fulfill all expectations right away. So the answer is no.
**Statement: "One must discard the prejudice that the truth must be something tangible."**	
I agree with the statement. The pursuit of truth often involves abstract concepts, intricate theories, and intangible ideas that cannot always be physically measured or touched. Truth in areas like philosophy, mathematics, and theoretical physics, for example, often transcends tangible reality. So the answer is yes.	[No such answer after repeatedly sampling 100 times.]
**Statement: "People open a window when they feel hot."**	
The statement "People open a window when they feel hot" is generally true for many individuals. Opening a window can allow for better air circulation and cooling, which can help alleviate the feeling of heat. However, it is important to note that this action may not apply to everyone or in every situation. For example, people in an air-conditioned environment or those with allergies might choose other methods to cool down. So while the statement holds in many cases, it is not universal. So the answer is yes	The statement "People open a window when they feel hot" is generally true but not universally applicable. Many people do open windows to cool down, leveraging the outside breeze for ventilation. However, there are other methods people might use to cool off, such as turning on a fan, adjusting the air conditioning, or using other cooling devices. Additionally, the effectiveness of opening a window might depend on external factors like the outside temperature or air quality. So the answer is no.
**Statement: "There is nothing more tragic in life than the utter impossibility of changing what you have done."**	
[No such answer after repeatedly sampling 100 times.]	While the statement captures the deep sense of regret and the weight of irreversible actions, it may be too absolute to say that there is nothing more tragic. Life's tragedies can take many forms, including loss, suffering, and missed opportunities. However, the inability to change past actions can indeed be profoundly painful and can lead to lasting emotional distress. But other experiences and circumstances in life can be equally, if not more, tragic depending on one's perspective. So the answer is no.

Table S9. GPT-4o's ratings of some statements, in response to question (a) "Do you agree with this statement?" We ask the model to give its reasoning first, followed by its binary answer at the end. Some answers are not observed after repeatedly sampling 100 times.

**(b) Do you think most people would agree with this statement? Please explain what you think in a few sentences, then end our answer with "So the answer is yes." or "So the answer is no."**	
**Statement: "The proliferation of charter schools affects the ability of states to efficiently distribute tax dollars."**	
The statement suggests that the growth of charter schools influences how states allocate their tax revenue. Many people might agree with this statement because charter schools often divert funding from traditional public schools, leading to complexities in budget distribution. Additionally, the funding formulas and accountability measures for charter schools can differ, causing further strain on state education budgets and financial planning. Given these factors, it's reasonable to conclude that the proliferation of charter schools does indeed impact the efficient distribution of tax dollars. So the answer is yes.	The statement "The proliferation of charter schools affects the ability of states to efficiently distribute tax dollars" is likely to generate mixed opinions. Some people might agree, arguing that charter schools create a parallel education system that can divert funds away from traditional public schools, leading to inefficient distribution of resources. Others might disagree, believing that charter schools introduce competition and innovation, potentially improving overall efficiency in education spending. Given this diversity of perspectives, it's unclear if most people would agree with the statement. So the answer is no
**Statement: "One must discard the prejudice that the truth must be something tangible."**	
It is likely that many people would agree with the statement because truth is often understood as a concept that can encompass abstract ideas, principles, or even subjective experiences, which are not necessarily tangible. Philosophical and scientific communities, in particular, frequently deal with truths that are conceptual rather than physical. This broader understanding of truth allows for a richer and more nuanced appreciation of knowledge and reality. So the answer is yes.	The statement suggests that truth can transcend physical or concrete forms, potentially encompassing abstract concepts, emotions, or beliefs. While some people may resonate with this idea, considering truths can be found in philosophical, mathematical, or ethical domains, many others might struggle with accepting intangible truths due to a preference for empirical evidence and tangible validation. Overall, society's varied perspectives on truth mean that not everyone will agree with this statement. So the answer is no.
**Statement: "Test your ideas follow the evidence and always remember that you could be wrong."**	
I think most people would theoretically agree with the statement, as it embodies principles of critical thinking, scientific inquiry, and humility. Many value the pursuit of truth and understand the importance of being open to changing one's mind based on new evidence. However, in practice, personal biases, emotions, and cognitive dissonance often lead individuals to stick to their preconceptions. So while the ideal is widely accepted, adherence varies. So the answer is yes.	I think that while many people might agree with the sentiment behind the statement, not everyone consistently applies it in practice. Humans often hold onto their beliefs and ideas tightly due to cognitive biases, emotional attachments, and social influences. Although the statement promotes a rational and open-minded approach to thinking, in reality, not everyone is always willing or able to rigorously test their ideas and embrace the possibility of being wrong. So the answer is no.
**Statement: "If you want to take finals then you should attend a class."**	
The statement "If you want to take finals then you should attend a class" implies a conditional relationship between attending classes and taking finals. Many educational systems and institutions require students to attend classes to be eligible to take the finals as this ensures that students have adequately received the instruction needed to succeed. This policy also promotes attendance and participation in the learning process. Given that this is a common understanding in educational contexts, most people likely agree with the statement. So the answer is yes.	The statement "If you want to take finals then you should attend a class" implies that attending class is a prerequisite for taking finals. While it may be a common expectation in many educational settings to attend class regularly in order to be eligible for finals, this is not universally enforced. Some institutions may allow students to take finals regardless of attendance, provided they meet other requirements such as coursework and registration. Therefore, it is not certain that most people would agree with the statement. So the answer is no

Table S10. GPT-4o's ratings of some statements, in response to question (b) "Do you think most people would agree with this statement?" We ask the model to give its reasoning first, followed by its binary answer at the end. Some answers are not observed after repeatedly sampling 100 times.

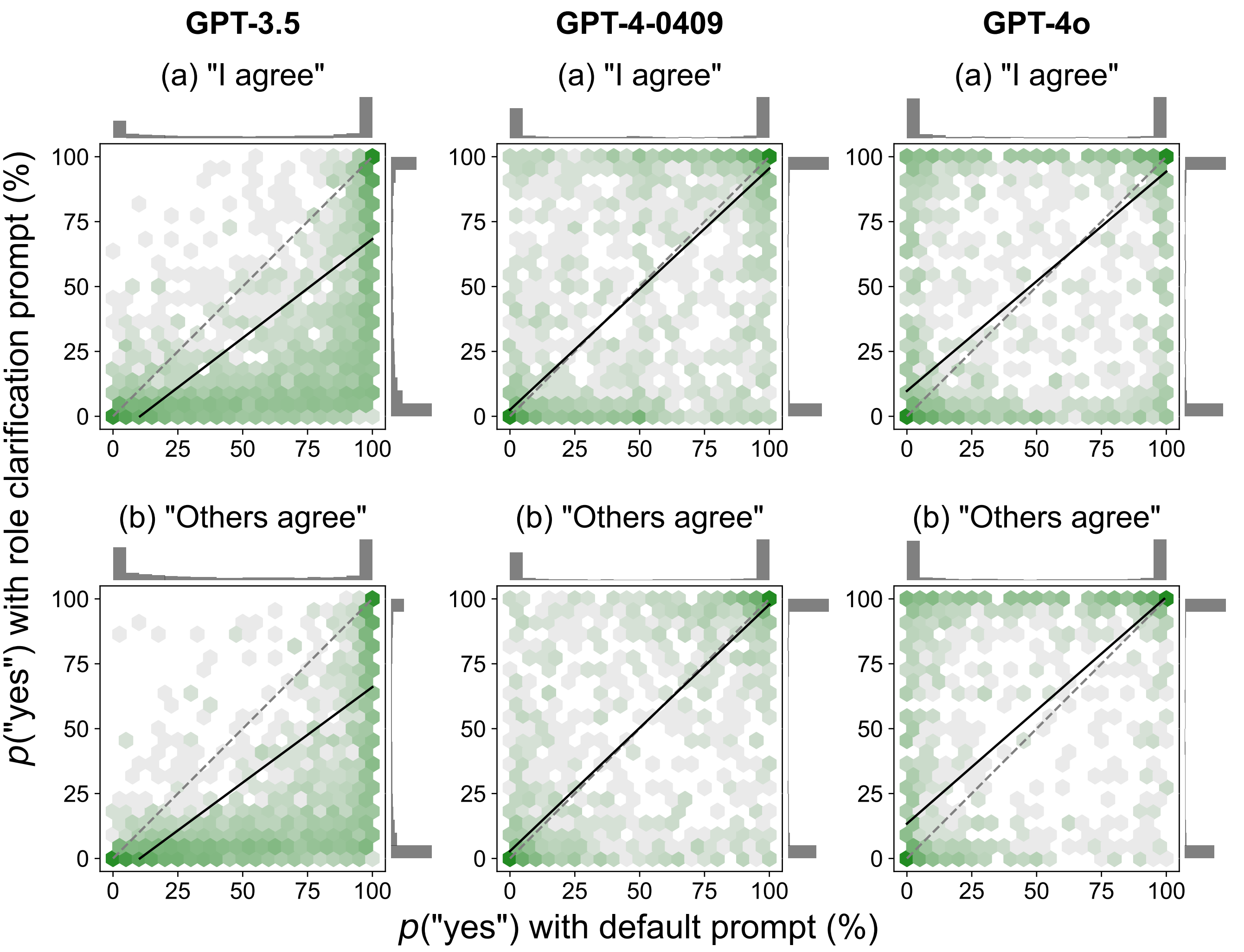

Figure S9. Calibration between a model's probabilities in two settings: without system prompt and with system prompt. The model is given a statement, and asked two questions: (a) "Do you agree with this statement?" and (b) "Do you think most people would agree with this statement?" The x-axis is the probability of the model's answer to the default prompts. The y-axis is the same probability but to the role clarification prompts. This is to explicitly discourage the model from answering *only* to satisfy the user. Also illustrated are the marginal distributions, the perfect calibration line (dashed black) and the best-fit line (solid black).

**Individual-level common sense (M_m, %)**					
GPT-3.5		GPT-4-0409		GPT-4o	
Default	*Role clarification*	*Default*	*Role clarification*	*Default*	*Role clarification*
76.8	63.7	80.6	82.1	75.2	81.3
**Statement-level common sense (Pearson correlation with humans)**					
GPT-3.5		GPT-4-0409		GPT-4o	
Default	*Role clarification*	*Default*	*Role clarification*	*Default*	*Role clarification*
.33	.09	.24	.20	.13	.20

Table S11. Evaluation results of GPT-3.5, GPT-4-0409 and GPT-4o under two prompting modes: default and role clarification.

Default prompt	Role clarification prompt
**Statement: "One must discard the prejudice that the truth must be something tangible."**	
**(a) Do you agree with this statement?**	
Yes. The statement encourages us to broaden our understanding of truth beyond the confines of physical tangibility. While tangible evidence and sensory experiences are crucial in many contexts, especially in science and daily life, they do not encompass the entirety of what can be considered true. For example, mathematical truths, logical principles, and abstract concepts such as justice or love are not tangible, yet they hold significant validity and impact. Additionally, philosophical and metaphysical inquiries often deal with truths that are not accessible through sensory	Yes. Truth can take many forms and is not always something tangible or physical. It can be abstract, conceptual, subjective, or based on personal experiences and beliefs. Discarding the prejudice that truth must be tangible allows for a more open and inclusive understanding of different kinds and sources of truth.
**(b) Do you think most people would agree with this statement?**	
No. While some people might agree with the statement, especially those with a philosophical or abstract outlook on truth, many others might find it challenging to accept that truth is not necessarily tangible. In general, people often rely on tangible evidence and concrete experiences to verify and understand truths. This is deeply ingrained in scientific thinking and everyday reasoning, where physical proof and observable data play crucial roles. Therefore, while the sentiment encourages open-mindedness and the consideration of abstract or conceptual truths, it may not resonate with	No. Most people tend to equate truth with something that can be observed, measured, or proven, which means they often see truth as something tangible or concrete. This belief is deeply rooted in the way we are taught to understand the world through empirical evidence and facts. However, some people may recognize that truth can also be abstract or conceptual, but they are likely in the minority.
**Statement: "Starting a new job people do what is expected of them for that job."**	
**(a) Do you agree with this statement?**	
Yes. Starting a new job typically involves meeting the expectations set by the employer. New employees often try to understand and fulfill their roles and responsibilities to make a positive impression and secure their position. They might be motivated to demonstrate their capabilities, align with the company's goals, and adhere to the prescribed norms and procedures. However, this doesn't mean that every individual will perfectly meet these expectations from the start. There can be a learning curve, and the extent to which a new employee successfully meets job expectations can vary	Yes. When starting a new job, people generally aim to meet the expectations set by their employer and fulfill the responsibilities of their role. This is often driven by a desire to make a good impression, succeed in the job, and maintain employment. However, while this is typically true, individual behavior can vary based on personal values, work ethic, and understanding of job requirements.
**(b) Do you think most people would agree with this statement?**	
Yes, I think most people would agree with the statement that when starting a new job, people generally do what is expected of them for that job. This is because new employees are typically motivated to make a good impression, want to succeed in their new roles, and often feel a sense of obligation or responsibility to meet the expectations outlined by their employers. Additionally, during the initial period of any job, there is usually a training or onboarding process that clearly communicates the tasks, responsibilities, and performance standards expected	Yes. Most people would probably agree with this statement because it is generally expected that individuals will perform the duties and responsibilities associated with their new job to meet the expectations of their employer and to make a good initial impression.

Table S12. Continuation in GPT-4o's ratings of some statements. The left-hand column contains responses to our original, default prompts. The right-hand column contains responses to the role clarification prompts. This is to explicitly discourage the model from answering *only* as to satisfy the user. Some answers are not finished because we set a 100-token limit on generation.

F Analyzing Commonsensicality for Various Types of Statements

In this section, we present some analysis of human and LLM common sense with respect to different kinds of statements in our corpus. In particular, we use every epistemological dichotomy defined in Section D.1, and separate the corpus into two complementary subsets, such as facts *vs.* opinions. Then, we calculate the individual commonsensicality score of every human participant and every LLM similar to the description in Section C, *but only with respect to the focal subset of statements*, such as only facts.

Table S13 on page 35 presents individual commonsensicality scores calculated for humans and LLMs in 13 settings: "all," which includes all $n_s = 4,407$ statements and 12 other settings, which include 2 subsets arising from each of the 6 dichotomies. The choice of feature can have a sizable influence on a model's (and human's) score. For example, in nearly all cases, this score increases the most if its calculation is restricted to statements that depict a fact or are a description of physical reality. Interestingly, focusing on figures of speech seems to improve the commonsensicality scores of Claude 3 Haiku, Flan-T5-Small, and LLaMA-2-13B/70B the most.

Model	All	Fa	Op	Ph	Soc	Lit	FoS	Pos	No	K	R	Ev	Abs
Claude 3 Haiku	61.4	61.7	61.2	63.8	60.2	59.5	**69.2**	65.5	59.8	61.9	61.1	59.9	63.9
Claude 3 Sonnet	61.5	64.1	60.1	66.6	59.0	60.9	64.0	**67.6**	59.2	63.4	60.4	60.1	64.0
Claude 3 Opus	75.4	78.3	73.8	**79.1**	73.6	75.5	74.9	78.2	74.3	75.7	75.2	75.4	75.4
DBRX	76.3	79.9	74.2	**80.5**	74.2	76.4	75.6	79.8	74.9	78.0	75.3	76.5	75.9
Falcon-7B	66.3	72.6	62.7	67.6	65.7	68.6	57.0	68.6	65.5	**76.3**	60.3	69.3	61.2
Falcon-40B	75.1	**80.1**	72.1	79.8	72.7	75.6	72.8	79.8	73.3	79.2	72.5	75.7	74.0
Falcon-180B	79.9	**85.4**	76.8	83.2	78.3	81.4	73.8	82.6	78.9	83.2	77.9	81.3	77.5
Flan-T5-Small	34.2	28.0	37.7	33.1	34.7	31.9	**43.7**	31.9	35.0	24.1	40.3	31.3	39.1
Flan-T5-Base	58.1	57.5	58.5	58.6	57.9	59.2	53.8	57.4	58.4	55.6	59.7	**60.2**	54.6
Flan-T5-Large	76.9	**81.1**	74.5	80.2	75.2	78.4	70.6	80.7	75.4	79.5	75.3	78.0	75.1
Flan-T5-XL	73.0	**76.7**	70.9	76.2	71.4	74.1	68.2	76.5	71.7	75.6	71.4	73.9	71.5
Flan-T5-XXL	80.4	82.8	79.0	**83.1**	79.1	82.1	73.3	82.3	79.7	81.7	79.6	81.9	77.8
Gemma-2B	65.9	69.5	63.8	65.8	65.9	67.6	58.5	67.4	65.3	**71.6**	62.4	67.7	62.8
Gemma-7B	72.0	**76.5**	69.5	75.7	70.2	72.2	71.3	75.4	70.8	75.1	70.2	73.2	70.1
Gemini Pro 1.0	79.7	**83.9**	77.3	83.7	77.7	80.8	75.2	83.2	78.4	82.0	78.3	80.2	78.9
GPT-3.5	76.8	79.7	75.2	**80.2**	75.1	77.1	75.7	79.4	75.9	78.8	75.7	76.9	76.7
GPT-4-0125	78.4	81.0	77.0	**81.4**	77.0	78.5	78.2	80.9	77.5	80.4	77.2	78.5	78.3
GPT-4-0409	80.6	82.9	79.3	**83.1**	79.3	80.8	79.7	82.6	79.8	82.9	79.1	80.8	80.2
GPT-4o	75.2	76.2	74.6	77.4	74.1	75.1	75.6	**77.6**	74.2	77.2	73.9	74.0	77.1
GPT-5	75.7	77.8	74.5	**79.4**	73.8	75.6	76.1	78.5	74.6	77.2	74.8	74.7	77.3
LLaMA-2-7B	75.0	**77.1**	73.8	76.0	74.5	76.0	70.6	76.9	74.3	77.1	73.7	75.2	74.6
LLaMA-2-13B	46.5	40.5	49.9	46.3	46.6	44.6	**54.5**	46.1	46.6	42.7	48.8	43.6	51.5
LLaMA-2-70B	63.5	63.9	63.4	66.5	62.1	62.6	**67.6**	67.4	62.1	65.8	62.2	61.7	66.7
LLaMA-3-8B	61.7	61.4	61.8	64.2	60.4	60.8	65.3	65.0	60.4	63.4	60.6	59.2	**66.0**
LLaMA-3-70B	74.4	76.7	73.0	**77.6**	72.7	74.5	73.7	76.8	73.4	76.1	73.3	73.8	75.3
Mistral-7B	80.4	**84.0**	78.4	83.1	79.1	82.1	73.6	83.3	79.4	83.9	78.4	81.7	78.3
Mixtral-8x7B	76.4	79.3	74.7	**80.2**	74.5	76.6	75.6	79.9	75.0	79.4	74.5	76.0	77.1
Mixtral-8x22B	82.3	**86.2**	80.2	85.8	80.6	83.3	78.5	85.0	81.3	84.8	80.8	83.0	81.1
Mistral-Large	81.3	84.6	79.4	**85.7**	79.1	81.9	78.6	84.7	80.0	84.5	79.3	81.4	81.0
OLMo-7B	72.7	**79.2**	68.9	76.0	71.0	74.9	63.5	76.2	71.3	78.7	69.0	75.2	68.4
Qwen2-0.5B	66.8	72.9	63.3	67.9	66.2	69.0	57.6	69.0	66.0	**76.2**	61.0	69.6	61.9
Qwen2-1.5B	74.6	78.0	72.7	77.1	73.3	76.0	68.9	76.9	73.7	**78.6**	72.1	75.7	72.6
Qwen2-7B	80.4	**83.8**	78.4	83.0	79.1	81.4	76.1	82.8	79.4	81.8	79.5	81.2	79.0
Qwen2-57B	80.9	**84.2**	79.0	83.4	79.7	81.6	78.2	82.4	80.3	82.9	79.7	82.2	78.7
Qwen2-72B	81.1	84.8	79.1	**85.2**	79.1	81.9	77.9	84.1	80.0	84.3	79.2	81.8	80.1
Human median	70.1	**78.5**	66.0	78.1	66.5	72.2	63.0	78.2	67.5	76.3	67.0	72.7	67.1

Table S13. Individual commonsensicality (%) of LLMs and humans. The "All" column contains commonsensicality scores calculated with respect to all 4,407 statements in the corpus. Every other column, from "Fa" to "Abs," contains the same scores but only with respect to statements that have the corresponding epistemological feature. "Fa": fact, "Op": opinion, "Ph": physical, "Soc": social, "Lit": literal language, "FoS": figure of speech, "Pos": positive, "No": normative, "K": knowledge, "R": reasoning, "Ev": everyday, and "Abs": abstract. These features are defined in Section D.1. Note that the human median is not directly comparable to every model's score, because every human only labeled 50 randomly chosen statements, whereas every model labels all statements. The highest score on every row is in bold.

SI References

1. M. E. Whiting, D. J. Watts, A Framework for Quantifying Individual and Collective Common Sense. *Proceedings of the National Academy of Sciences* **121**(4): e2309535121 (2024).
2. S. Baron-Cohen, S. Wheelwright, J. Hill, Y. Raste, I. Plumb, The "Reading the Mind in the Eyes" Test Revised Version: A Study with Normal Adults, and Adults with Asperger Syndrome or High-functioning Autism. *Journal of Child Psychology and Psychiatry* **42**(2): 241–251 (2001).
3. M. Bialek, G. Pennycook, The Cognitive Reflection Test Is Robust to Multiple Exposures. *Behavior Research Methods* **50**(5): 1953–1959 (2018).
4. M. Sap, *et al.*, Atomic: An Atlas of Machine Commonsense for If-Then Reasoning. *Proceedings of the AAAI Conference on Artificial Intelligence* **33**(1): 3027–3035 (2019).
5. Anthropic, The Claude 3 Model Family: Opus, Sonnet, Haiku. (2024). https://www-cdn.anthropic.com/de8ba9b01c9ab7cbabf5c33b80b7bbc618857627/Model_Card_Claude_3.pdf (Accessed 2024-05-28).
6. Mosaic, Introducing DBRX: A New State-of-the-Art Open LLM. (2024). https://www.databricks.com/blog/introducing-dbrx-new-state-art-open-llm (Accessed 2024-06-15).
7. E. Almazrouei, *et al.*, The Falcon Series of Open Language Models. arXiv [Preprint] (2023). https://doi.org/10.48550/arXiv.2311.16867 (Accessed 2024-01-01).
8. H. W. Chung, *et al.*, Scaling Instruction-Finetuned Language Models. arXiv [Preprint] (2022). https://doi.org/10.48550/arXiv.2210.11416 (Accessed 2023-04-05).
9. Google, Gemma: Open Models Based on Gemini Research and Technology. (2024). https://storage.googleapis.com/deepmind-media/gemma/gemma-report.pdf (Accessed 2024-02-03).
10. Google, Gemini 1.5: Unlocking Multimodal Understanding across Millions of Tokens of Context. (2024). https://storage.googleapis.com/deepmind-media/gemini/gemini_v1_5_report.pdf (Accessed 2024-03-01).
11. T. Brown, *et al.*, Language Models Are Few-Shot Learners. *Advances in Neural Information Processing Systems* **33**: 1877–1901 (2020).
12. OpenAI, *et al.*, GPT-4 Technical Report. arXiv [Preprint] (2023). https://arxiv.org/abs/2303.08774 (Accessed 2024-03-20).
13. OpenAI, GPT-4o System Card. (2024). https://cdn.openai.com/gpt-4o-system-card.pdf (Accessed 2024-08-01).
14. OpenAI, GPT-5 System Card. (2025). https://cdn.openai.com/pdf/8124a3ce-ab78-4f06-96eb-49ea29ffb52f/gpt5-system-card-aug7.pdf (Accessed 2025-08-19).
15. H. Touvron, *et al.*, Llama 2: Open Foundation and Fine-Tuned Chat Models. arXiv [Preprint] (2023). https://doi.org/10.48550/arXiv.2307.09288 (Accessed 2023-09-12).
16. A. Grattafiori, *et al.*, The Llama 3 Herd of Models. arXiv [Preprint] (2024). https://arxiv.org/abs/2407.21783 (Accessed 2025-01-06).
17. A. Q. Jiang, *et al.*, Mistral 7B. arXiv [Preprint] (2023). https://arxiv.org/abs/2310.06825 (Accessed 2023-11-13).
18. A. Q. Jiang, *et al.*, Mixtral of Experts. arXiv [Preprint] (2024). https://doi.org/10.48550/arXiv.2401.04088 (Accessed 2024-06-07).
19. MistralAI, Cheaper, Better, Faster, Stronger. (2024). https://mistral.ai/news/mixtral-8x22b/ (Accessed 2024-06-15).
20. MistralAI, Au Large. (2024). https://mistral.ai/news/mistral-large/ (Accessed 2024-05-28).
21. D. Groeneveld, *et al.*, "OLMo: Accelerating the Science of Language Models" in *Proceedings of the 62nd Annual Meeting of the Association for Computational Linguistics (Volume 1: Long Papers)* (2024), pp. 15789–15809.
22. A. Yang, *et al.*, Qwen2 Technical Report. arXiv [Preprint] (2024). https://doi.org/10.48550/arXiv.2407.10671 (Accessed 2024-12-11).
23. W.-L. Chiang, *et al.*, "Chatbot Arena: An Open Platform for Evaluating LLMs by Human Preference" in *Proceedings of the 41st International Conference on Machine Learning* (2024).
24. T. Mihaylov, P. Clark, T. Khot, A. Sabharwal, "Can a Suit of Armor Conduct Electricity? A New Dataset for Open Book Question Answering" in *Proceedings of the 2018 Conference on Empirical Methods in Natural Language Processing* (2018), pp. 2381–2391.
25. P. Liang, *et al.*, Holistic Evaluation of Language Models. *Transactions on Machine Learning Research* (2023).
